# Molecular basis for hierarchical histone de-β-hydroxybutyrylation by SIRT3

**DOI:** 10.1038/s41421-019-0103-0

**Published:** 2019-07-09

**Authors:** Xingrun Zhang, Ruili Cao, Jinrong Niu, Shumin Yang, Huida Ma, Shuai Zhao, Haitao Li

**Affiliations:** MOE Key Laboratory of Protein Sciences, Beijing Advanced Innovation Center for Structural Biology, Beijing Frontier Research Center for Biological Structure, Tsinghua-Peking Joint Center for Life Sciences, Department of Basic Medical Sciences, School of Medicine, Tsinghua University, 100084 Beijing, China

**Keywords:** X-ray crystallography, Histone post-translational modifications

## Abstract

Chemical modifications on histones constitute a key mechanism for gene regulation in chromatin context. Recently, histone lysine β-hydroxybutyrylation (Kbhb) was identified as a new form of histone acylation that connects starvation-responsive metabolism to epigenetic regulation. Sirtuins are a family of NAD^+^-dependent deacetylases. Through systematic profiling studies, we show that human SIRT3 displays class-selective histone de-β-hydroxybutyrylase activities with preference for H3 K4, K9, K18, K23, K27, and H4K16, but not for H4 K5, K8, K12, which distinguishes it from the Zn-dependent HDACs. Structural studies revealed a hydrogen bond-lined hydrophobic pocket favored for the S-form Kbhb recognition and catalysis. β-backbone but not side chain-mediated interactions around Kbhb dominate sequence motif recognition, explaining the broad site-specificity of SIRT3. The observed class-selectivity of SIRT3 is due to an entropically unfavorable barrier associated with the glycine-flanking motif that the histone Kbhb resides in. Collectively, we reveal the molecular basis for class-selective histone de-β-hydroxybutyrylation by SIRT3, shedding lights on the function of sirtuins in Kbhb biology through hierarchical deacylation.

## Introduction

Posttranslational modifications (PTMs) on histones, often installed, recognized, and removed by their cognate “writers, readers, and erasers” in a type-specific and site-specific manner, play critical roles in regulating diverse chromatin-templated cellular processes^1,2^. Histone lysine acetylation (Kac) was firstly identified in early 1960s^3^, possessing a primary function in regulating gene transcription^4,5^. Extensive studies on the dynamic regulation and selective recognition of histone Kac have elucidated its role in various cellular regulatory mechanisms^6,7^. Aided by advanced mass spectrometry-based proteomics technologies, a cornucopia of non-acetyl histone acylations have been identified, such as formylation (fo)^8^, propionylation (pr), butyrylation (bu)^9,10^, crotonoylation (cr)^11^, succinylation (succ)^12^, glutarylation (glu)^13^, 2-hydroxyisobutyrylation (hib)^14^, and β-hydroxybutyrylation (bhb)^15^. Identification of writers, readers, and erasers of these acyl marks are important to further elucidate the chromatin signaling pathways in which they are involved^16,17^.

Histone lysine β-hydroxybutyrylation (Kbhb) has been detected in yeast, fly, mouse and human, and 44 Kbhb sites have been identified in human and mouse cells^15^. β-hydroxybutyrylation modified on the ε-group of lysine distinguishes itself from acetylation by its branch, chiral, and four-carbon length properties. The levels of histone Kbhb are significantly elevated under the conditions of starvation or streptozotocin-induced diabetic ketosis. It has been proposed that histone Kbhb directly connects ketone body metabolism to gene regulation, given the high concentration of β-hydroxybutyrate in blood during fasting, starvation, or prolonged intense exercise^15,18^. Importantly, histone H3K9bhb is associated with gene upregulation in a starvation-responsive manner, and distinguishes an amount of these genes from others that are marked by H3K9ac and H3K4me3, suggesting a unique role in connecting epigenetic regulation and starvation-responsive metabolism^15^. To further elucidate Kbhb functions in gene regulation, its cognate eraser(s) awaits to be characterized.

The sirtuin family proteins (e.g., human SIRT1-7), a class of NAD^+^-dependent deacetylases that remove acetyl marks from various cellular proteins including histones, function in a wide range of biological pathways in responses to nutritional and environmental perturbations^19^. Activation of sirtuins increases the lifespan of several model organisms, while mutation or inhibition of sirtuins leads to the onset of aging phenotypes^20,21^. Recently, emerging evidence suggested that sirtuins could remove acyl groups other than acetyl from lysine residues^22^. For example, SIRT1-3 can remove crotonyl marks from lysine^23^, while SIRT4 is reported to have deglutarylation and dehydroxymethyl-glutarylation avtivities^24,25^. SIRT5 removes the malonyl, succinyl, and glutaryl marks^13,26,27^. SIRT6 preferentially hydrolyzes long alkyl acyl marks from lysines, such as myristoyl mark^28^. In summary, sirtuins play an important role in removing various acyl marks from lysines of histone as well as non-histone substrates.

SIRT3 is a predominant mitochondria matrix protein that modulates the activity of key metabolic enzymes via protein deacetylation^29^. Besides, several studies reported that SIRT3 could act as a histone deacylase both in vitro and in vivo^23,30–32^, suggesting a moonlighting function of SIRT3 in nucleus^33^. Through systematic profiling studies, here we reported that human SIRT3 functions as a histone de-β-hydroxybutyrylase at sites H3 K4, K9, K18, K23, K27, and H4K16. Interestingly, SIRT3 is incapable of removing bhb at sites H4 K5, K8, K12 that have flanking glycine residues both in vitro and in cells. Such class-selectivity is not observed in HDAC3, a key member of the Zn-dependent histone deacetylases. Co-crystal structural analyses of SIRT3 bound to H3K9bhb, H3K4bhb, and H4K16bhb peptides as well as structural-based mutagenesis studies revealed the molecular basis underlying class-selective recognition and erasure of histone Kbhb. Hence, our work on hierarchical histone deacylation by SIRT3 suggests a potential regulatory mechanism that links acylation dynamics to gene regulation under metabolic alternations.

## Results

### Systematic profiling histone deacylase activities of sirtuins

**Fig. 1:**
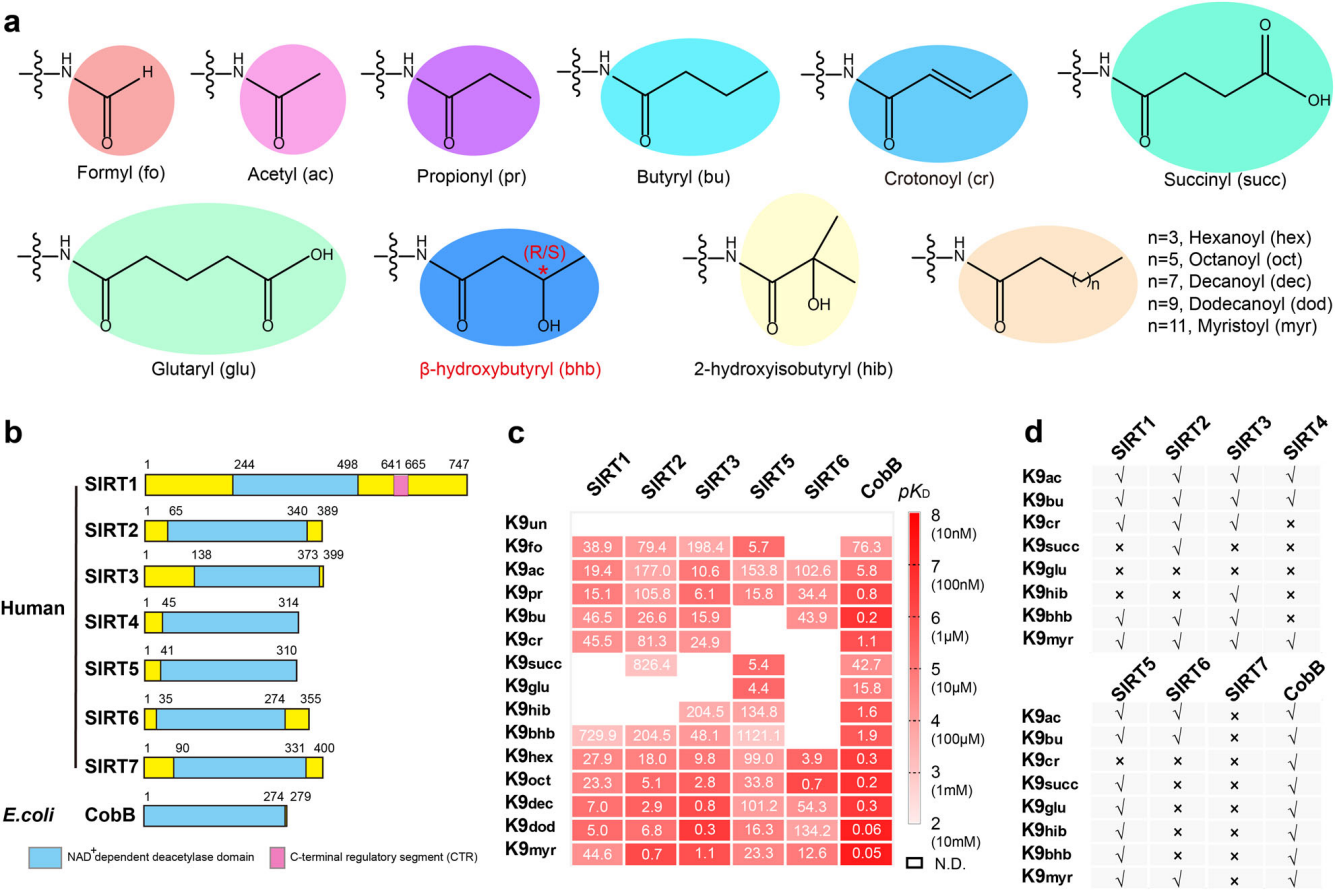
Deacylase activity profiling of sirtuins. **a** Chemical structures of major lysine acylations. Abbreviations of each acylation mark are used throughout this report. **b** Domain architectures of human sirtuins and *E. coli* CobB. NAD^+^-dependent deacetylase domains are colored in cyan. **c** Affinity heatmap between histone H3_1–15_K9 acylation peptides and sirtuin proteins. The binding *K*_D_s ranging 10^−2^–10^−8^ mol/L were transformed into values ranging 2–8 via a -log*K*_D_ function, and expressed as a linear gradient of red color with annotated affinities. The binding *K*_D_s are noted at micromole level in the panel. See also Supplementary Table S1; Fig S2. **d** Summary of deacylation activities catalyzed by human and *E. coli* sirtuin proteins. The enzymatic activity is revealed by MALDI-TOF mass spectrometry (MS). See also Supplementary Fig S3. Note: no evidences for Khex, Koct, Kdec, Kdod, Kmyr on endogenous histones have been reported so far and these acylations are synthesized for in vitro study only

By means of click chemistry followed by affinity purification and competition assays, Bao et al revealed that SIRT3 is a histone decrotonylase and binds to H3K4 crotonylation (H3K4cr) peptide substrate at an affinity of ~25.1 μM in the absence of NAD^+^. Taking histone H3K9 acylation as a probe, we synthesized a panel of histone H3 peptides encompassing residues 1–15 (H3_1–15_) with lysine 9 bearing acetyl and thirteen other non-acetyl acyl marks (Fig. 1a). Next, we expressed and purified NAD^+^-dependent deacetylase domains of all human sirtuins as well as full-length bacteria CobB that serves as a prototype sirtuin control (Fig. 1b and Supplementary Fig. S1). We then performed isothermal titration calorimetry (ITC) to profile interactions between the well-behaved sirtuin proteins (SIRT1-3, 5, 6, and CobB) and histone H3K9 acylation peptides in the absence of NAD^+^. The yields of SIRT4 and SIRT7 were low and thereby were not used for ITC titration.

On the binding affinity heatmap of ninety interaction pairs, each sirtuin family member displays unique H3K9 acylation binding feature (Fig. 1c and Supplementary Fig. S2; Table S1). Previously reported specific deacylase activities of sirtuins are all corroborated by micromolar binding affinities in this heatmap, with *K*_D_ values of 24.9 µM for SIRT3-Kcr, of 5.4 and 4.4 µM for SIRT5-Ksucc and SIRT5-Kglu, and of 12.6 µM for SIRT6-Kmyr (Fig. 1c)^13,23,26,28^. Most sirtuins bind well to straight-chain acyl marks from 1-carbon Kfo to 14-carbon Kmyr, except that SIRT5 does not bind Kbu/Kcr, and SIRT6 does not bind Kfo/Kcr. The two acidic acyl marks, namely Kglu and Ksucc, are recognized by SIRT5 and CobB; interestingly, Ksucc but not Kglu is accommodated by SIRT2. The hydroxyl-replaced Khib and Kbhb marks are both recognized by SIRT3, SIRT5, and CobB; however, only Kbhb but not Khib can be recognized by SIRT1 and SIRT2 (Fig. 1c). The diversified acyllysine selectivity reflects delicate design of the active center over a common sirtuin scaffold. It is interesting to note that most sirtuins displayed optimal binding towards non-acetyl acyl marks, notably those with longer alkyl chain (Fig. 1c and Supplementary Fig. S2; Table S1).

Next, we measured deacylase activities of all sirtuins including SIRT4 and SIRT7 by mass spectrometry (MS) against eight H3K9 acylation marks: Kac, Kbu, Kcr, Ksucc, Kglu, Khib, Kbhb, and Kmyr. In general, all binding events mentioned above were well translated into deacylase activities (Fig. 1d and Supplementary Fig. S3). This is consistent with the notion that sirtuin enzymes preferentially bind to acylated peptide first before NAD^+^ loading^34^, and efficient peptide substrate engagement is required for deacylation to occur. Collectively, bacterial CobB is able to deacylate eight H3K9 acylation marks tested but SIRT7 does none of them. SIRT4 and SIRT6 are able to hydrolyze Kac, Kbu, and Kmyr but not the acidic Ksucc/Kglu, the hydroxyl-replaced Khib/Kbhb, and the rigidified Kcr marks. In contrast, SIRT5 is able to remove most marks except for Kcr. SIRT1-3 behave similarly and are sensitive to acidic Ksucc/Kglu, except for SIRT2-Ksucc; additionally, SIRT1 and SIRT2 cannot hydrolyze Khib, while SIRT3 displays an deacylase activity towards both Khib and Kbhb (Fig. 1d).

### Enzymatic characterization of SIRT3 as an H3K9bhb eraser

**Fig. 2:**
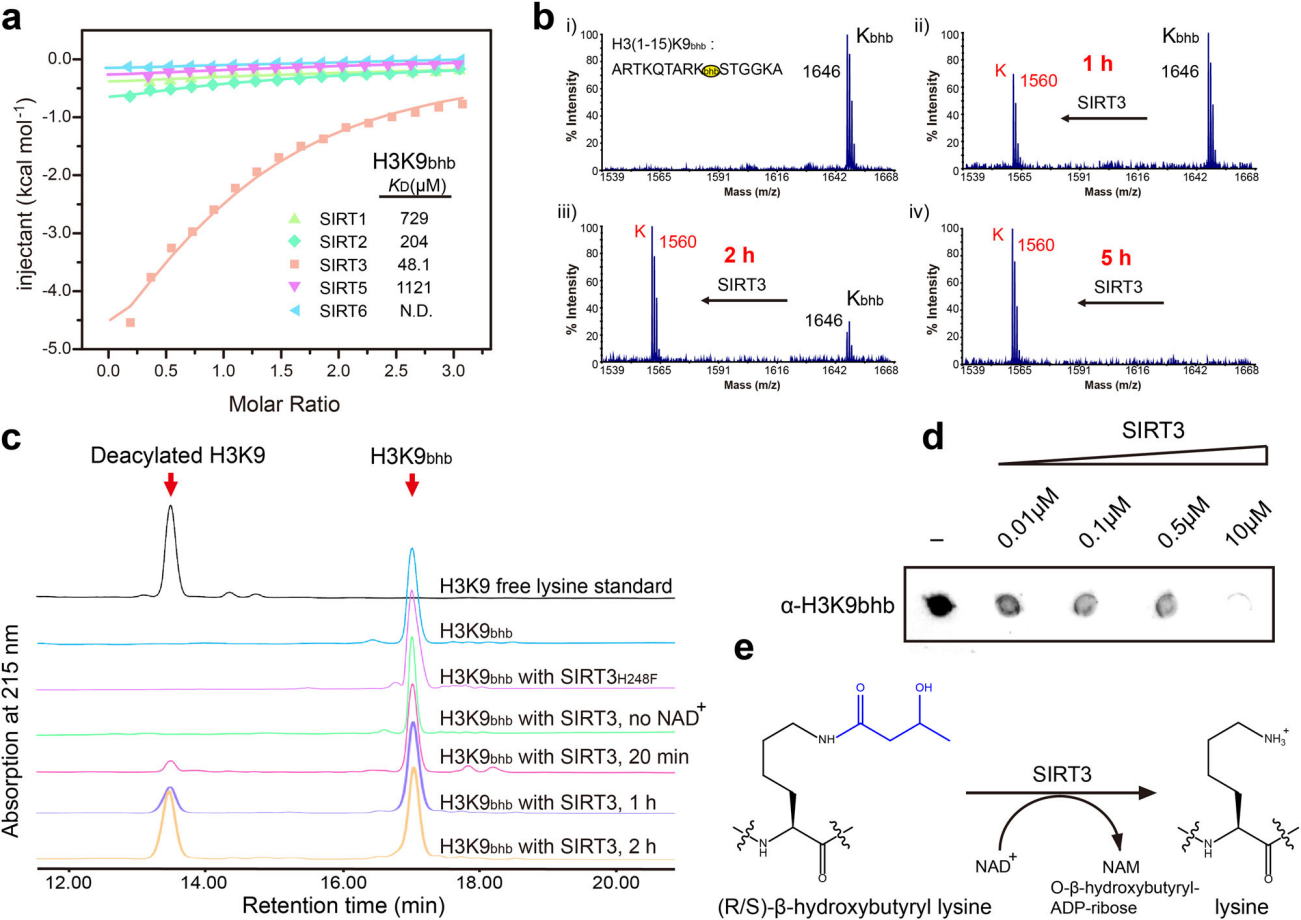
Human SIRT3 is an efficient H3K9bhb eraser. **a** ITC fitting curves of several human sirtuin proteins titrated with H3_1-15_K9bhb peptide. N.D. not detected. **b**–**d** MALDI-TOF MS (**b**), RP-HPLC (**c**), and dot-blot (**d**) based deacylation assays of SIRT3 with H3K9_1–15_K9bhb peptide as substrate. **e** Schematic diagram of the de-β-hydroxybutyrylation reaction catalyzed by SIRT3

Since SIRT3 showed strongest affinity for H3K9bhb among human sirtuins (SIRT3, *K*_D_ = 48.1 μM; SIRT1, *K*_D_ = 729 μM; SIRT2, *K*_D_ = 204 μM; SIRT5, *K*_D_ = 1121 μM; SIRT6, N.D.) (Fig. 2a), and SIRT4 and SIRT7 displayed no enzymatic activity towards H3K9bhb (Fig. 1d), we next chose SIRT3 for in-depth enzymatic and structural characterizations. MS, RP-HPLC (reverse-phase high performance liquid chromatography) and dot-blot-based assays were used to probe the H3K9bhb deacylase activity of SIRT3 (Fig. 2b–d). As expected, SIRT3 removes the bhb group from H3K9bhb in a time, enzyme-dose, and NAD^+^ cofactor dependent manner, demonstrating an NAD^+^-dependent de-β-hydroxybutyrylation activity (Fig. 2e). Mutation of residue H248 (H248F) that is critical for the catalysis of deacetylation by SIRT3 completely abolished its de-β-hydroxybutyrylation activity (Fig. 2c), indicating that SIRT3 catalyzes Kbhb hydrolysis by a similar mechanism as it does for Kac^35^.

### Overall structure of SIRT3 bound to H3K9bhb peptide

**Fig. 3:**
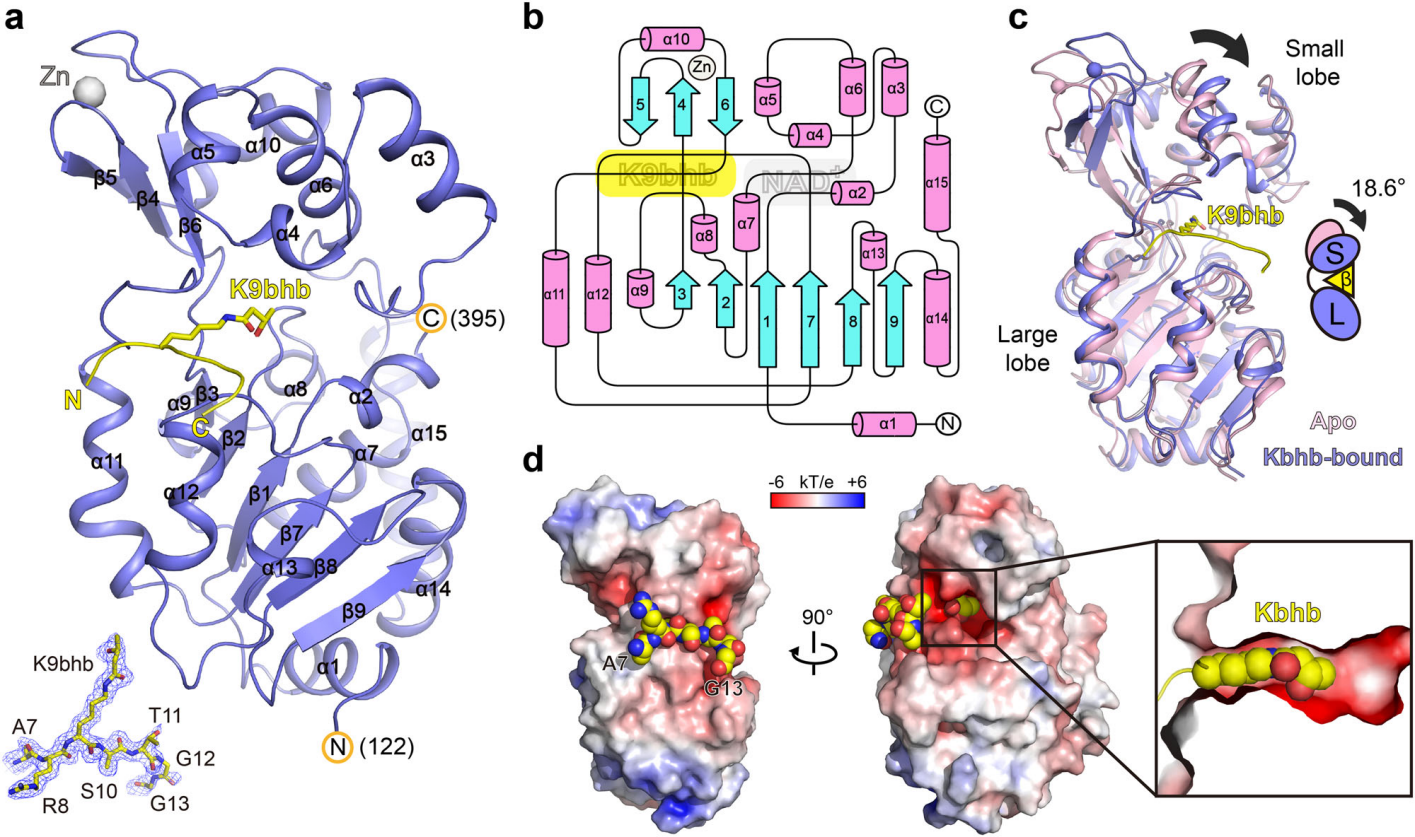
Overall structure of SIRT3-H3K9bhb complex. **a** Ribbon view of SIRT3 (blue) bound to H3_6–15_K9bhb peptide (yellow) with K9bhb highlighted as sticks. Gray ball, zinc ion of the zinc finger motif within SIRT3. Residues A7 to G13 of the H3_6–15_K9bhb peptide are well traced as shown by the Fo-Fc omit map countered at 2.2 σ level. **b** The topology of SIRT3 catalytic domain. Magenta cylinders, α-helices; Cyan arrows, β-strands. Binding positions of zinc, H3K9bhb and NAD^+^ cofactor were annotated as gray circle, yellow and gray rectangles, respectively. **c** Conformational changes of SIRT3 upon H3K9bhb peptide binding. H3K9bhb-bound binary structure (blue) is superimposed with the apo form SIRT3 (pink, PDB: 3GLS). Note the 18.6° rotation of the small lobe as illustrated in the schematic diagram. **d** Electrostatic surface view of SIRT3 bound to H3K9bhb peptide depicted as space-filling spheres. Electrostatic potential is expressed as a spectrum ranging from −6 kT/e (red) to +6 kT/e (blue). A cutaway view of the Kbhb insertion pocket is highlighted in the close-up box. Note the extra space next to the tip of Kbhb

To decipher the structural basis underlying H3K9bhb recognition by SIRT3, we determined the binary crystal structure of SIRT3_118–399_ bound to the H3_6–15_K9bhb peptide in an NAD^+^ free state at 1.95 Å resolution (Supplementary Table S2). There is one SIRT3 monomer in one crystallographic asymmetric unit. Based on the electron density, we could model residues 122–395 of SIRT3 and trace the “_7_ARKSTGG_13_” segment of the H3 peptide (Fig. 3a). SIRT3 adopts a classical sirtuin fold that consists of a zinc-binding small lobe and a NAD^+^-binding large lobe arranged in a Rossmann fold (Fig. 3b). The histone peptide (and NAD^+^) binds to a cleft formed at the interface of the two lobes. Structural alignment of the binary complex with an apo form SIRT3 (PDB: 3GLS) revealed induced bending of the small lobe upon H3K9bhb peptide insertion (Fig. 3c), consistent with previous report^35^. We calculated a rotation angle of 18.6° based on the DynDom3D webserver analysis^36^. The peptide-binding surface is largely negatively charged, being electrostatically favorable for the basic histone peptide targeting (Fig. 3d, left). There is a snug fitting of the H3K9bhb peptide into the substrate-binding pocket with Kbhb side chain inserted into an elongated tunnel (Fig. 3d, right). Consistent with the observed preference of SIRT3 for long-chain acylations, extra space exists next to the bhb group, being well positioned to accommodate long-chain acyl marks like Kdod and Kdec (Fig. 1c).

### Interaction details for H3K9bhb recognition by SIRT3

**Fig. 4:**
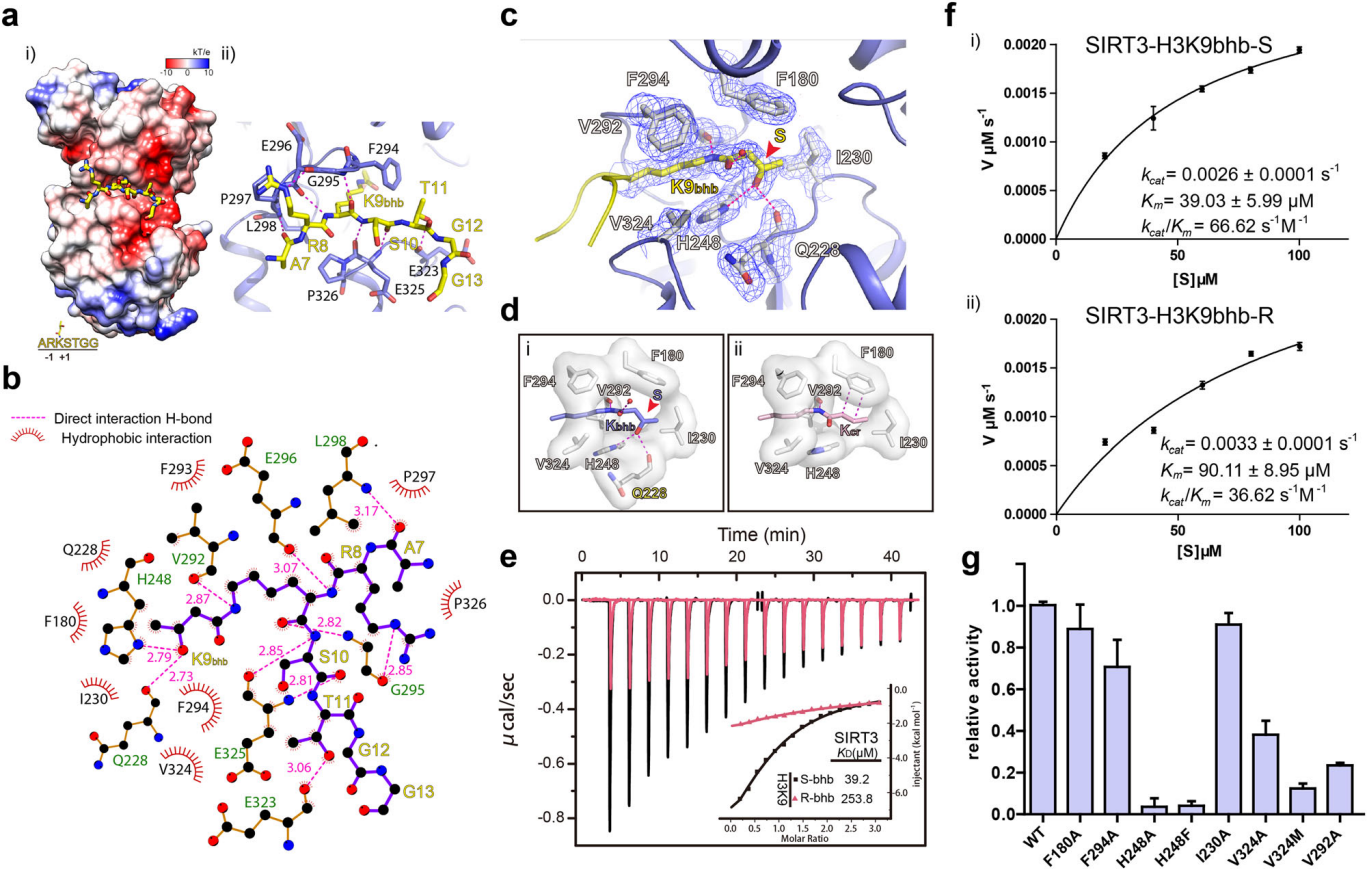
Molecular and biochemical characterization of SIRT3-mediated H3K9bhb recognition and catalysis. **a** Insertion of H3K9bhb peptide (yellow sticks) into an electronegative surface of SIRT3 (i) and hydrogen-bonding network for H3K9 peptide recognition (ii). SIRT3 is represented in surface mode and colored as a spectrum of its electrostatic potential ranging from −10 kT/e (red) to +10 kT/e (blue). Dashed lines, hydrogen bonds. **b** LIGPLOT diagram listing critical contacts between the H3K9bhb peptide and SIRT3. H3 segment (purple) and key residues of SIRT3 (brown) are depicted in ball-and-stick mode. Black ball, carbon; blue ball, nitrogen; red ball, oxygen; magenta dash line, direct interaction hydrogen bond. **c** H3K9bhb mark (yellow sticks) recognition within the Kbhb-binding pocket. Blue meshes, Fo-Fc omit map countered at the 2.2 σ level. Key pocket residues are shown as gray sticks. Red arrow denotes the chiral center of bhb and an S-form enantiomer was observed. Small red ball, water molecule. **d** Comparison of the recognition modes of Kbhb (i) and Kcr (ii) by SIRT3. Coordinates of the SIRT3-Kcr complex was taken from the PDB_REDO entry 4V1C. Magenta dashes denote either hydrogen bonding (i) or π–π stacking (ii) interactions. **e** ITC fitting curves of SIRT3 titrated with H3K9bhb-S/R peptides. **f** Michaelis-Menten plots of enzymatic kinetics of H3K9bhb-S(i)/R(ii) catalyzed by SIRT3. **g** In vitro deacylation assays comparing wild-type and mutant SIRT3. H3_1–15_K9bhb peptide was used as substrate and RP-HPLC was adopted to measure the yield of the de-β-hydroxybutyrylated product by peak integration. Error bars represent standard deviation of three repeats

In the binary structure of SIRT3 bound to H3K9bhb, the backbone of H3 segment “A7-R8-K9-S10-T11” engages five hydrogen bonding pairs, including A7_(CO)_:L298_(NH)_, K9_(NH)_:E296_(CO)_, K9_(CO)_:G295_(NH)_ from the upper side and S10_(NH)_:E325_(CO)_, S10_(CO)_:E325_(NH)_ from lower side (Fig. 4a, b). These are further strengthened by additional H3 side-chain mediated hydrogen bonds involving R8:G295 (upper) and T11:E323 (lower) (Fig. 4a, b).

The Kbhb mark is inserted into an elongated pocket formed by residues F294, V292, F180, I230, Q228, H248, and V324 (Fig. 4c). Besides an effect of charge neutralization of lysine, the bhb moiety of Kbhb affords extra hydrophobicity and hydrogen-bond forming capacity. Compliantly, the Kbhb mark is recognized by both hydrophobic contacts involving residues F294, V324, F180, I230, and hydrogen bonding interactions involving residues Q228, H248, V292 and a molecule of water (Fig. 4c). SIRT3 can deacylate both Kbhb and Kcr marks. As revealed in previous structural studies^23^, the π–π stacking between F180 and Kcr plane as well as hydrophobic contacts contribute to Kcr recognition (Fig. 4dii). The saturated bhb moiety cannot form π–π stacking with F180. As an adaptation, hydrophobic contacts with F180 and I230, as well as direct hydrogen bonds of the β-hydroxyl group with H248 and Q228 contribute to bhb-specific mode of recognition (Fig. 4di).

It has not escaped our attention that both electron density (Fig. 4c) and interaction environment (Fig. 4di) clearly suggest that the S-form enantiomer of bhb is captured in the crystal structure despite the fact that the Kbhb peptide was synthesized as the R/S racemic mixture. In support, ITC titration using newly synthesized stereospecific H3K9bhb peptides revealed a ~6.5-fold binding preference for S-bhb (*K*_D_ = 39.2 μM) over R-bhb (*K*_D_ = 253.8 μM) (Fig. 4e). Consistently, enzymatic kinetic assays revealed a *K*_m_ of 39.03 μM for S-bhb and a *K*_m_ of 90.11 μM for R-bhb, and in total ~2-fold catalytic efficiency preference for the S-form (*k*_cat_/*K*_m_ = 66.62 s^−1^ M^−1^) over the R-form (*k*_cat_/*K*_m_ = 36.62 s^−1^ M^−1^) enantiomers (Fig. 4f). Our structural analyses revealed that the S-form bhb is concurrently stabilized by hydrogen bonding and hydrophobic contacts. By contrast, in the case of R-form Kbhb, the substrate engagement mode is imperfect since the abovementioned hydrogen bonding interactions and the hydrophobic contacts are not compatible (Supplementary Fig. S4a).

### Catalytic center and mutagenesis-based enzymatic studies

In order to validate the functional importance of the pocket residues, we generated corresponding mutants of SIRT3_118–399_ and measured their deacylase activity by RP-HPLC. As shown in Fig. 4g, mutation of the catalytic residue H248 (H248A and H248F) almost completely abrogated the activity, attesting to its essential role in catalysis and/or substrate recognition. F180A, I230A, V292A, F294A, and V324A are also detrimental to the hydrolysis of Kbhb by SIRT3 to various degrees. We designed another mutant, residue V324M, intended for blocking insertion of the Kbhb moiety into its binding pocket. This mutant, retaining only about 15% activity of the wild-type enzyme, is most severe in inhibiting the hydrolysis of Kbhb by SIRT3 besides the H248 mutants (Fig. 4g). The V324M mutant is about 14-fold lower in binding affinity with K9bhb than wild-type SIRT3 (SIRT3_WT_-H3K9_bhb_, *K*_D_ = 48.1 μM; SIRT3_V324M_-H3K9_bhb_, *K*_D_ = 664 μM) possibly due to the blocking of Kbhb insertion into the binding pocket as we designed (Supplementary Fig. S4b). These results demonstrate the important roles of these residues in recognition and hydrolysis of Kbhb by SIRT3.

### Kbhb site-selectivity of SIRT3 over H3 and H4 N-terminal tails in vitro

**Fig. 5:**
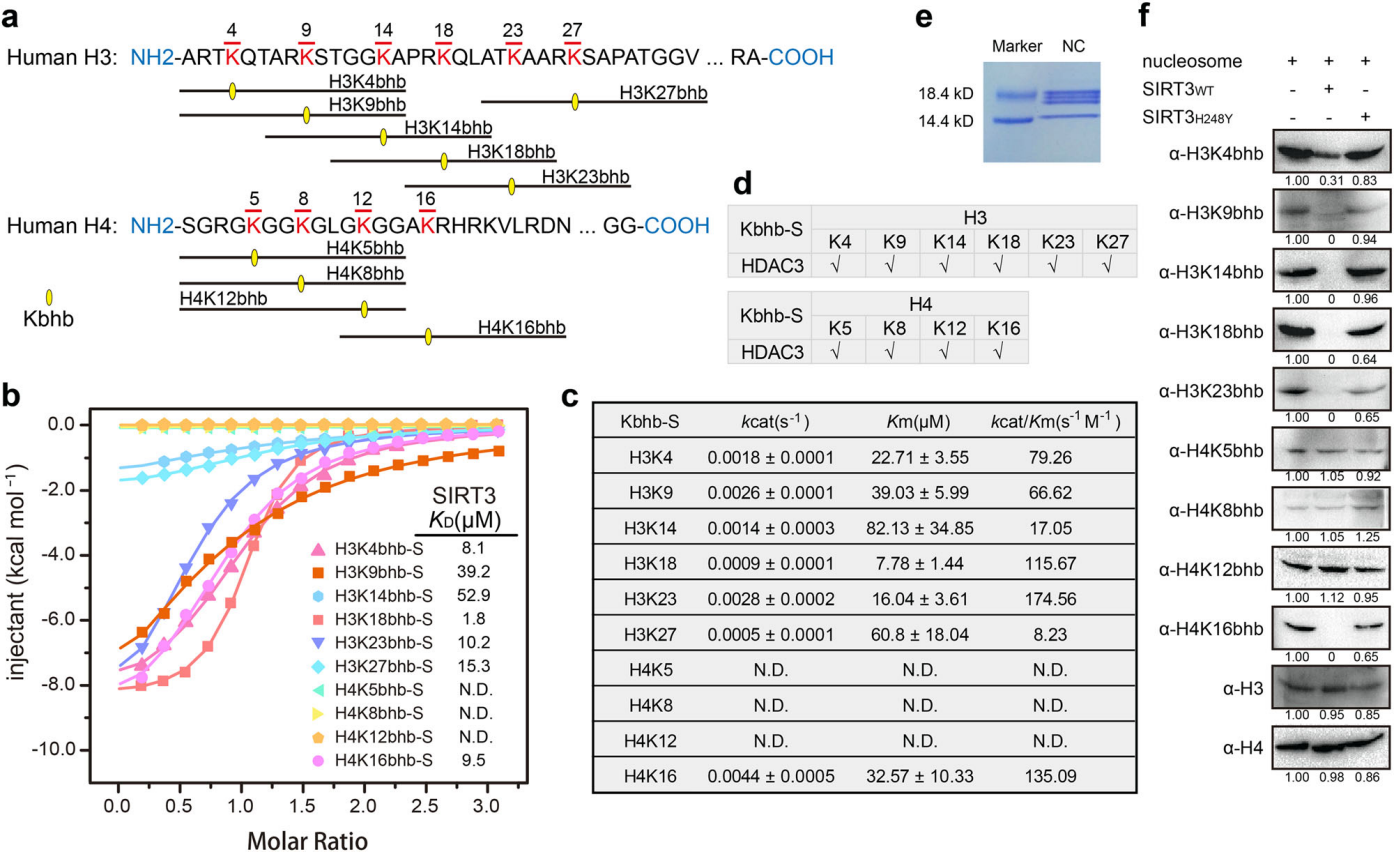
Class-selective de-β-hydroxybutyrylation of histones H3 and H4 by SIRT3 in vitro. **a** Schematic diagram of histone Kbhb peptides used in ITC and deacylation assays. **b** ITC fitting curves of SIRT3 titrated with all histone S-form Kbhb peptides listed in **a**. **c** Schematic diagram of enzymatic kinetics parameters of histone Kbhb-S peptides catalyzed by SIRT3. N.D. not detected. **d** Summary of de-β-hydroxybutyrylation activities over all histone S-form Kbhb peptides listed in **a** catalyzed by HDAC3 (measured by MALDI-TOF MS). **e** Coomassie blue staining results of extracted nucleosomes from HEK293T cells. NC, nucleosome. **f** Immunoblotting results of deacylation assays on Kbhb-modified nucleosome by both wild type and catalytic mutant SIRT3 (SIRT3_H248Y_). Histone H3 and H4 were used as loading controls

To investigate the site-selectivity for recognition and hydrolysis of Kbhb by SIRT3, we tested whether SIRT3 can engage and remove bhb mark on well-known acylation sites of histones, including K4, K9, K14, K18, K23, K27 of H3, and K5, K8, K12, K16 of H4 (Fig. 5a). In ITC assays, SIRT3 broadly binds to Kbhb mark with affinities 8.1 μM for H3K4bhb, 39.2 μM for H3K9bhb, 52.9 μM for H3K14bhb, 1.8 μM for H3K18bhb, 10.2 μM for H3K23bhb, 15.3 μM for H3K27bhb, and 9.5 μM for H4K16bhb in the S-form (Fig. 5b), which displayed ~2.3 to 7.6-fold binding preference over the R-form (Supplementary Fig. S5a). By contrast, SIRT3 does not bind to K5bhb, K8bhb, and K12bhb of H4 in both chiral forms, and thus displays a unique class-selectivity against these sites. Consistently, we observed broad de-β-hydroxybutyrylase activities of SIRT3 on tested histone sites other than H4 K5, K8, and K12 in RP-HPLC based deacylation assays (Fig. 5c and Supplementary Figs. S5b, S6). The enzymatic kinetics results show a relatively broad range of *k*_cat_/*K*_m_ (79.26 s^−1^ M^−1^ for H3K4bhb-S, 66.62 s^−1^ M^−1^ for H3K9bhb-S, 17.05 s^−1^ M^−1^ for H3K14bhb-S, 115.67 s^−1^ M^−1^ for H3K18bhb-S, 174.56 s^−1^ M^−1^ for H3K23bhb-S, 8.23 s^−1^ M^−1^ for H3K27bhb-S, and 135.09 s^−1^ M^−1^ for H4K16bhb-S), which is consistent with ITC binding assays. The glycine-flanking motifs shared by H4K5bhb (GKGG), K8bhb (GGKG), and K12bhb (GKGG) probably account for the enzymatic incompetence of SIRT3. In support, H3K14bhb that has two flanking glycine residues from the N-terminus (GGKA) displayed weakest binding affinity (Fig. 5b and Supplementary Fig. S5a). In comparison to SIRT3, the class I Zn-dependent histone deacetylase HDAC3 showed no class-selectivity on histone H3 and H4 tails (Fig. 5d nd Supplementary Figs. S5b, S7), highlighting a molecular functional distinction between the two subfamilies of histone deacylases.

To test if SIRT3 could deacylate Kbhb at nucleosomal level, we prepared Kbhb modified nucleosomes from bhb-treated HEK293 cell and subjected them for deacylation by SIRT3. Dot blot assays verified the site-selectivity and type-selectivity of corresponding antibodies (Supplementary Fig. S8). After SIRT3 treatment, we immunoblotted Kbhb of the SDS-PAGE resolved histone samples (Fig. 5e). We were able to detect clear deacylation activity of SIRT3 over H3K4bhb, H3K9bhb, H3K14bhb, H3K18bhb, H3K23bhb, and H4K16bhb, but not H4K5bhb, H4K8bhb, and H4K12bhb (Fig. 5f). Importantly, an active site mutant of SIRT3, H248Y, displayed no activity toward Kbhb-modified nucleosomes, suggesting a direct role of SIRT3 in nucleosomal deacylation of Kbhb.

Collectively, the above data demonstrate a class-selective de-β-hydroxybutyrylation activity of SIRT3 that distinguishes it from HDACs at both peptide and nucleosome levels in vitro.

### SIRT3 regulates histone Kbhb levels in nucleus

**Fig. 6:**
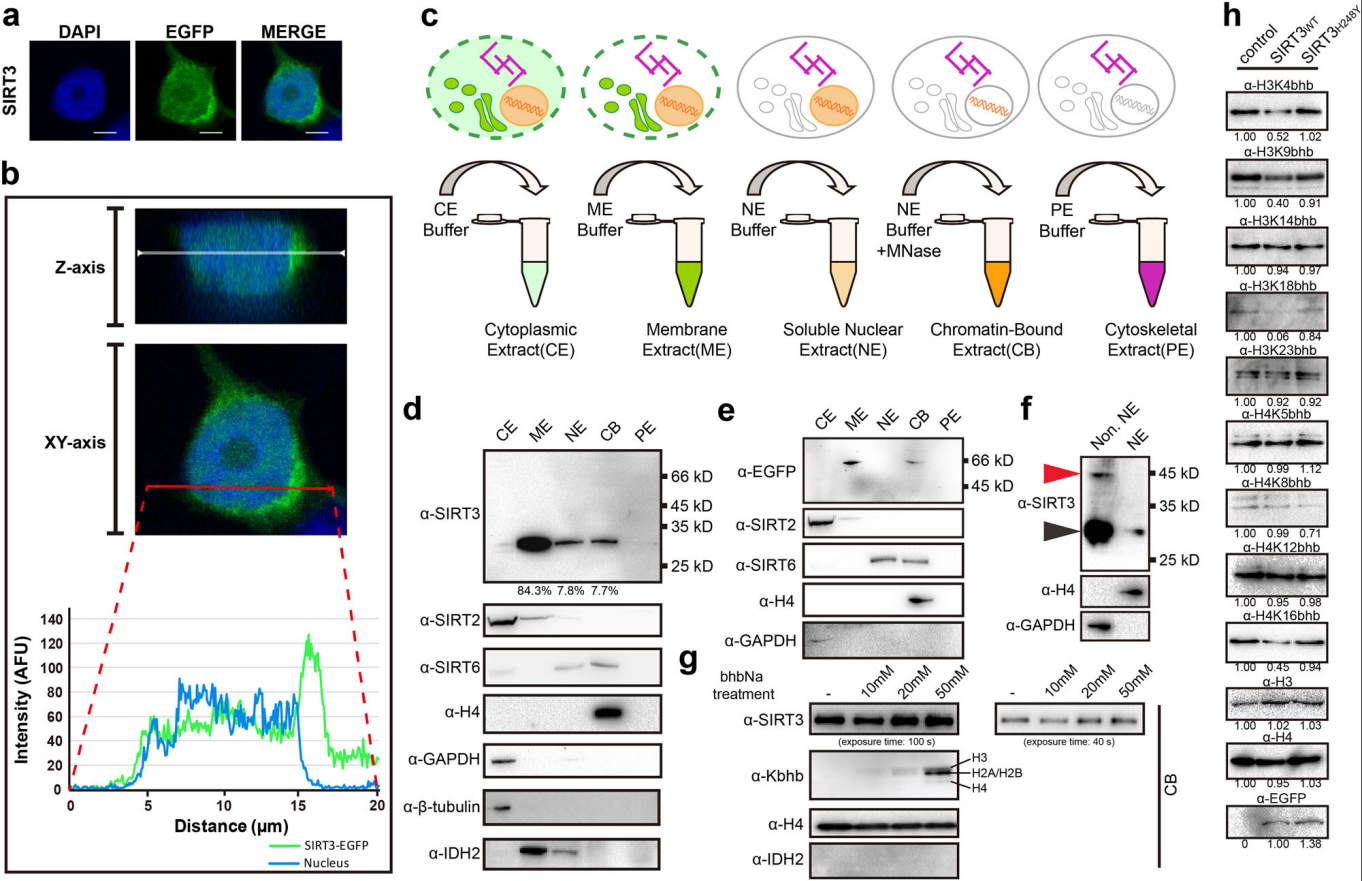
SIRT3 regulates histone Kbhb levels in a class-selective manner in nucleus. **a** Fluorescence analysis of SIRT3 in HEK293 cells. Scale bars, 5 μm. **b** A HEK293 cell was analyzed with DAPI (blue) and SIRT3-EGFP (green) and scanned at 0.38 μm increments along the *z* axis using confocal microscopy (top image). The fluorescence intensities midlane in the cell in the *XY* axis are shown (middle and bottom images). **c** Schematic diagram of subcellular fractionation procedures used in this assay (Thermo Scientific, CAT# 78840: Subcellular Protein Fractionation Kit for Cultured Cells). Note that all fractions are adjusted to the same volume before analysis to allow direct abundance comparison. **d**, **e** Western blots of HEK293 cell extracted fractions from wild-type cells (**d**) or after transfection with SIRT3-EGFP (**e**). CE, cytoplasmic extracts; ME, membrane extracts; NE, soluble nuclear extracts; CB, chromatin-bound extracts; PE, cytoskeletal extracts. **f** Immunoblotting results of full-length and short form SIRT3 in nuclear (NE) and none nuclear (Non. NE) fractions. **g** Immunoblotting results of SIRT3 levels after bhbNa treatment in chromatin-bound (CB) fractions. **h** Immunoblot of histone Kbhb levels of annotated marks in SIRT3 overexpressed HEK293 cells

SIRT3 is mainly a mitochondria matrix protein that regulates metabolism biochemically via protein deacetylation^29^. To confirm its nuclear presence, we performed fluorescence-based co-localization and cell fractionation studies in HEK293 cells. Z-stack 3D imaging of C-terminally EGFP-labeled SIRT3 revealed clear fluorescence signals in nucleus despite much brighter signals in mitochondria (Fig. 6a, b and Supplementary Fig. S9, Movies S1, S2). Subcellular fractionation assays showed that overexpressed or endogenous SIRT3 could be detected in both mitochondria and nuclear fractions (Fig. 6c–f). Notably, ~10% of total SIRT3 was observed in the chromatin-bound extraction. Immunoblotting assays using bhbNa treated cells showed that SIRT3 can be recruited onto chromatin in a dosage-dependent manner, possibly induced by upregulated histone Kbhb levels (Fig. 6g). Interestingly, while the Kbhb signal is enriched on histone H3 under lower and more physiological bhbNa concentrations (10 and 20 mM), the Kbhb levels of H2A/H2B are preferentially upregulated at a supplemented bhbNa concentration of 50 mM (Fig. 6g), suggesting mechanisms of histone type-specific Kbhb regulation in response to metabolic alternations. Collectively, all the above results suggest a direct association of SIRT3 with chromatin.

We further checked the deacylation activity in cells with overexpressed SIRT3. Consistently, overexpression of wild type but not the H248Y mutant SIRT3 reduced bhb levels at sites H3K4, H3K9, H3K18 and H4K16, but not H4K5, H4K8, H4K12 (Fig. 6h). Interestingly, the levels of H3K14bhb and H3K23bhb are not influenced at cellular level upon SIRT3 overexpression (Fig. 6h), which contradicts with a clear nucleosomal substrate activity in vitro (Fig. 5f), and suggests possible mechanisms of active establishment of H3K14bhb and H3K23bhb patterns in HEK293 cells. Of note, a “reading-and-writing” cross-talk between H3K14 and H3K23 acylations have been implicated in the case of MOZ/MORF (a.k.a. KAT6A/6B) complexes that are responsible for maintaining global histone H3K23ac level in a BRPF1-dependent manner^37^. We have previously shown that the DPF domains of MOZ and DPF2 are specific histone H3K14 acylation readers, and bind to H3K14bhb (racemic mixture) at 110 μM and 0.93 μM, respectively^38^.

### Recognitions of H3K4bhb and H4K16bhb by SIRT3

**Fig. 7:**
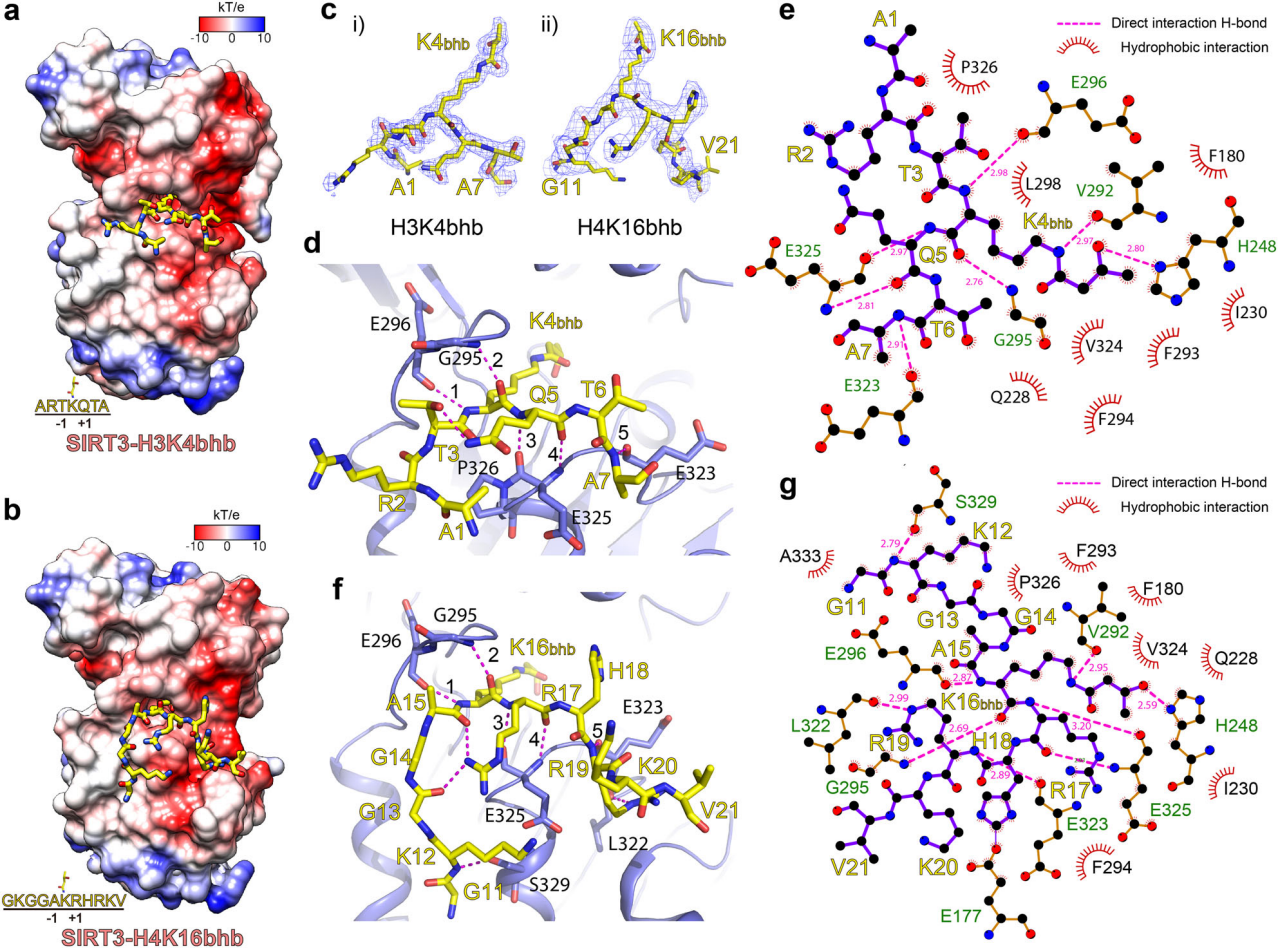
Structures of SIRT3 bound to H3K4bhb and H4K16bhb peptides. **a**, **b** Overall structures of SIRT3 bound to H3_1–15_K4bhb (**a**) or H4_11–25_K16bhb (**b**) peptides. SIRT3 is shown in electrostatic surface view; H3K4bhb and H4K16bhb peptides are shown as yellow sticks. **c** Fo-Fc omit maps (contoured 3.0 σ) of H3K4bhb and H4K16bhb peptides. Residues A1–A7 of H3K4bhb and G11-V21 of H4K16bhb were modeled. **d**, **f** Interaction details of SIRT3 with H3K4bhb (**d**) and H4K16bhb (**f**) peptides. SIRT3 is in blue ribbon representation with key residues shown as sticks. H3K4bhb or H4K16bhb peptides are shown as yellow sticks. Dashed lines, hydrogen bonds. **e**, **g** LIGPLOT diagrams listing contacts between SIRT3 and H3K4bhb (**e**) or H4K16bhb (**g**) peptides. The plotting strategy is the same as described in Fig. 4b

To compare consensus and difference in distinct histone Kbhb site recognition by SIRT3, we next solved the binary structures of SIRT3 bound to H3_1–15_K4bhb and H4_11–25_K16bhb peptides at 1.9 Å and 2.9 Å, respectively (Supplementary Table S2). Both H3K4bhb and H4K16bhb peptides are stapled into an acidic surface of SIRT3 with well-traced electron densities (Fig. 7a–c). The H3K4bhb peptide adopts the same orientation as that of H3K9bhb and the segment “A1-R2-T3-K4bhb-Q5-T6-A7” was modeled (Fig. 7d). Multiple hydrogen bonds and hydrophobic contacts contribute to H3K4bhb-SIRT3 interaction with the Kbhb mark recognized essentially the same way as K9bhb (Fig. 7e). In particular, H3K4bhb backbone engages five sets of hydrogen bonds, including K4_(NH)_:E296_(CO)_, K4_(CO)_:G295_(NH)_ from the upper side and Q5_(NH)_:E325_(CO)_, Q5_(CO)_:E325_(NH)_, A7_(NH)_:E323_(CO)_ from lower side (Fig. 7d, e). We also observed an intra-chain hydrogen bond between T3 and Q5 side chains, which may stabilize a bending conformation of the peptide backbone and thus facilitate H3K4bhb recognition.

The H4K16bhb peptide is recognized in a similar manner as H3K4bhb, which is through five sets of backbone-mediated hydrogen bonds and extensive hydrophobic contacts (Fig. 7f, g). Besides, H4K16bhb binding is further strengthened by two unique hydrogen bonding pairs involving distal flanking residues K12 (K12:S329) and R19 (R19:L322), as well as intra-chain hydrogen bonds involving R17 (R17:G13 and R17:A15) (Fig. 7f, g). These extra hydrogen-bonding interactions explained the relatively high binding affinity (H3K4bhb, *K*_D_ = 8.1 μM; H4K16bhb, *K*_D_ = 9.5 μM) compared with H3K9bhb recognition.

### Molecular basis underlying class-selectivity of SIRT3

**Fig. 8:**
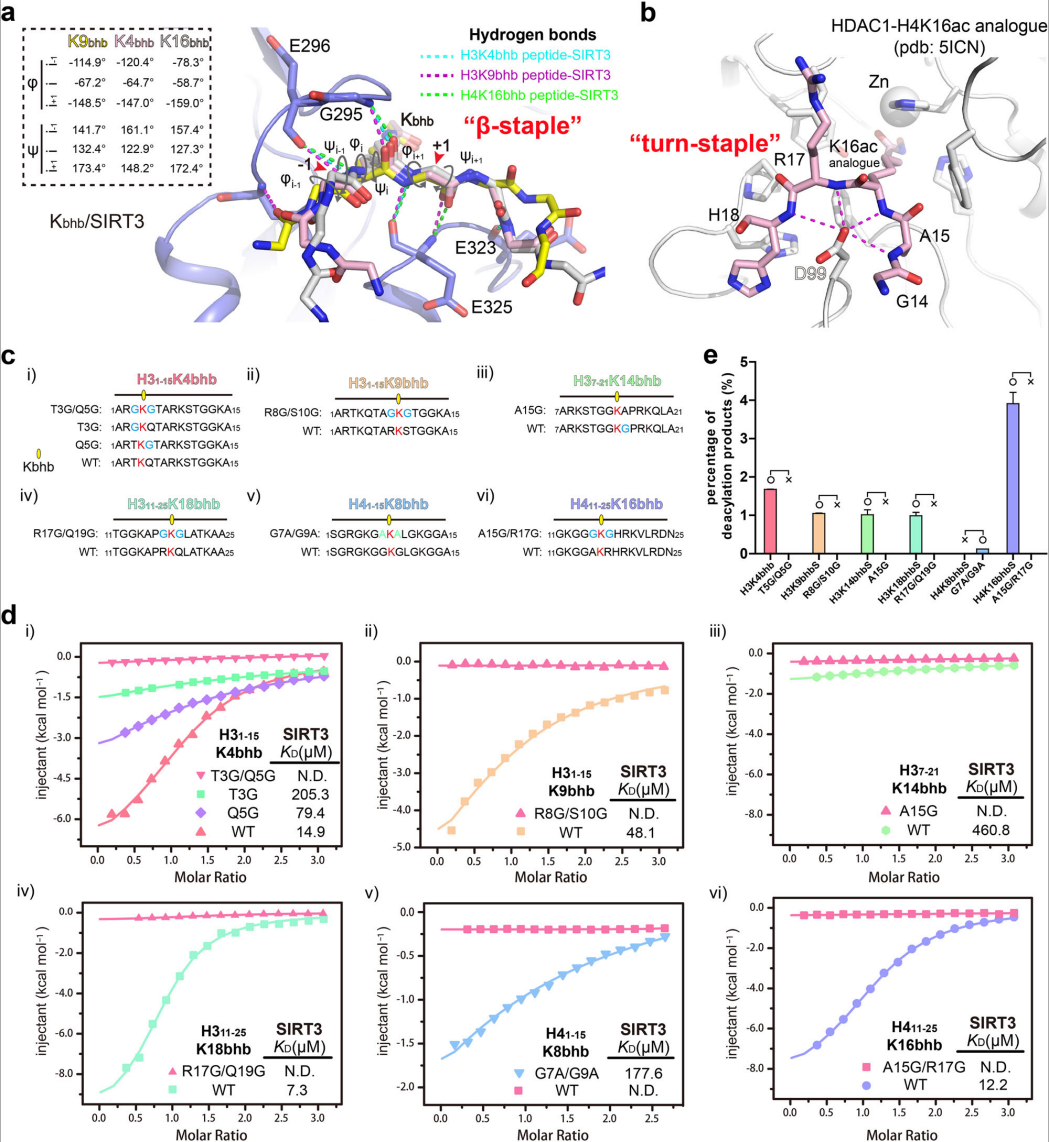
Molecular basis for class-selective histone de-β-hydroxybutyrylation by SIRT3. **a** β-staple recognition of H3K9bhb (yellow), H3K4bhb (pink) and H4K16bhb (gray) recognition by SIRT3. All three peptides are depicted as sticks and superimposed for comparison. For clarity, side chains of all peptides are omitted. Backbone dihedral angles (φ/ψ) are illustrated around the −1 to +1 positions of the Kbhb site, with values summarized on the top-left corner. Red arrows denote the Cα position of −1 and +1 residues. Dash lines, hydrogen bonds. **b** Backbone engagement for H4K16 recognition by HDAC1. Coordinates are taken from PDB entry 5ICN. The H4K16 was replaced by a hydroxamic acid functionality to mimic K16ac. Note that an acidic residue D99 dominates peptide backbone recognition in a non-β manner. **c** List of peptide sequences used for ITC titration. The Kbhb sites are highlighted red and its flanking glycine residues are highlighted blue. **d** ITC fitting curves of SIRT3 titrated with wild-type and glycine/alanine-mutant Kbhb peptides around histone sites H3K4 (i), H3K9 (ii), H3K14 (iii), H3K18 (iv), H4K8 (v) and H4K16 (vi). **e** In vitro deacylation assay results of all wild-type (WT) and mutant histone peptides listed in panel C catalyzed by SIRT3. Error bars represent standard error of mean of three repeats

Next, we aligned all three binary SIRT3 structures reported in this study, and found that four pairs of backbone interactions involving residues E296, G295, and E325 are highly conserved, which serves as a common binding mode and dominates Kbhb sequence motif recognition (Fig. 8a). Dihedral angle analysis revealed that main-chain geometries of the central Kbhb motif (*X*_-i_*K*_i_*X*_i+1_) fall in the core β-strand region with proper φ/ψ dihedral angle distribution (Fig. 8a)^39^. Interestingly, those sites that SIRT3 is incapable or inefficient to catalyze are characteristic of a glycine-flanking feature (Fig. 5a). Although the poor tolerance cannot be due to steric clash since glycine is side-chain free, the high degree of rotational freedom endowed by glycine could create an entropically unfavorable barrier for the abovementioned backbone interactions, thereby explaining the observed class-selectivity of SIRT3. In support, calorimetric titrations revealed a high entropy cost of −28.1 cal/mol/deg for H3K14bhb, which is in sharp contrast with an entropy change of −4.2 cal/mol/deg for H3K4bhb and −7.8 cal/mol/deg for H4K16bhb (Supplementary Table S1). Conceivably, the bending conformations of peptide substrate are pre-stabilized in part by intra-chain hydrogen bonding interactions in the cases of H3K4bhb and H4K16bhb (Fig. 7d, f).

Previous structural studies of HDAC1 (a close paralog of HDAC3) bound to an H4K16ac peptide analog suggested that class I HDACs adopt a deep and narrow pocket for acetyllysine insertion, and the histone peptide is organized in a “turn-staple” conformation around an acidic residue (D99 of HDAC1) for backbone engagement (Fig. 8b)^40^. Both SIRT3 and class I HDACs primarily rely on the substrate backbone for recognition, accounting for their relatively broad site-specificity. In the meantime, the backbone-binding mode of class I HDACs is not β-conformation selective, which is distinct from SIRT3 and therefore explains the observed non class-selectivity of HDAC3.

To further verify our analysis, we synthesized a series of mutant histone peptides by introducing or removing Kbhb-flanking glycine residues around representative sites including H3K4, H3K9, H3K14, H3K18, H4K8, H4K16 and subjected them for binding and enzymatic assays using SIRT3 (Fig. 8c). As summarized in Fig. 8d, single G mutation around H3K4bhb caused reduced binding by 15-fold for T3G (*K*_D_ = 205.3 μM) and by five-fold for Q5G (*K*_D_ = 79.4 μM), while “T3GQ5G” double mutation completely disrupted binding. Similarly, the existence of two Kbhb-flanking glycine residues at sites H3K9 (R8G/S10G), H3K14 (A15G), H3K18 (R17G/Q19G), and H4K16 (A15G/R17G) abolish interaction completely. In further support, reverse “G-to-A” mutation in the case of H4(G7A/G9A)K8bhb restored binding to a *K*_D_ of 177.6 μM (Fig. 8d). The results of enzymatic assays are consistent with our ITC binding assay results. All “GG” mutant peptides cannot be catalyzed by SIRT3, while “G-to-A” mutation in H4(G7A/G9A)K8bhb peptide rescues its deacylation ability by SIRT3 (Fig. 8e). Moreover, successful restoration of binding and catalysis by “G-to-A” mutation suggests that lack of charge/polarity in the “GG” motifs is unlikely an alternative reason for their being weak SIRT3 substrates.

Overall, our structural, binding and enzymatic studies demonstrate the molecular basis for anti-selective deacylation of glycine-flanking Kbhb sites of histones by SIRT3.

## Discussion

A large collection of non-acetyl acylations has been identified on histone lysines, posing new challenges to characterize the cognate regulators and regulatory mechanisms^16,41^. Lysine β-hydroxybutyrylation is unique as it possesses a hydroxyl-group and is chiral and structurally branched in nature (Fig. 1a). Through profiling studies, here we demonstrate that four human sirtuins (SIRT1-3, SIRT5), bacterial sirtuin CobB, and HDAC3 are able to catalyze the hydrolysis of histone Kbhb. Such newly identified de-β-hydroxybutyrylase activities broaden the landscape of histone PTMs that are regulated by sirtuins as well as class I HDACs. Importantly, the observed class-selectivity of SIRT3 but not HDAC3 provides new insights into a regulatory mechanism that histone Kbhb as well as other histone acylations likely involves through hierarchical histone deacylation by sirtuins.

Our structural studies revealed a hydrogen bond-lined hydrophobic pocket of SIRT3 for Kbhb recognition and catalysis. The recognition mode of Kbhb is different from that of Kcr (Fig. 4d). Besides a common hydrogen bond between V292 and the amide NH of Kbhb/Kcr, the bhb group is uniquely stabilized by hydrogen bonding of its β-hydroxyl with Q228 and H248 of SIRT3; additionally, F180 and I230 contribute to critical hydrophobic contacts with the hydrocarbon portion of bhb. Intriguingly, what is captured in the crystal structure is the S-form bhb, whose stereo configuration best permits the observed hydrophobic contact (Fig. 4c). Such a stereo-selectivity may have interesting functional implications given the fact that the R-β-hydroxybutyrate is the primary form of ketone body, while the S-form bhb-CoA is mainly converted from trans-cr-CoA via a hydration reaction especially during the short-chain fatty acids (SCFA) metabolism^42,43^. The prevalence of Kbhb stereo-specific modification and its regulation in cell remains to be explored in future studies. Our profiling studies also showed that another hydroxyl-substituted acyllysine, Khib, could be recognized and hydrolyzed by SIRT3, SIRT5, and CobB, but not SIRT1 and SIRT2 (Fig. 1a, c, d). The conservation and divergence of Khib versus Kbhb recognition by different sirtuin family members awaits further structural elucidations.

SIRT3 displayed both broad site-specificity (recognizing multiple acylation sites) and strict class-selectivity (unable to recognize a group of acylation sites) in histone de-β-hydroxybutyrylation. At the molecular level, these seemingly conflicting properties are well explained by a consensus β-staple selectivity of SIRT3^44^. As revealed by our parallel structural analyses, we showed that SIRT3 selects against glycine-flanking motifs due to their intrinsic flexibility. In addition to sites of H4K5, H4K8, H4K12 investigated here, other Kbhb sites of similar motifs are expected resistant to SIRT3 deacylation as well. As a key regulator in mitochondria, SIRT3-senstive mitochondrial acetylome has been profiled^45,46^. Remarkably, the “β-staple rule” is also conserved, in which flanking glycine or proline residues that are detrimental to the β-structure are disfavored^47,48^. Given the sequence and structural similarities of human sirtuins (Supplementary Fig. S10), the observed backbone selectivity is likely conserved among different sirtuin members or of different acylation types. This is supported by previous biochemical profiling studies using either acetyl-peptide microarray^46^ or pre-acylated (Kbhb, Khib not included) designer nucleosome library^49,50^, in which glycine-flanking Kac peptides or acylated H4 tail were shown to be poor substrates for all human sirtuins. HDAC3 also possesses histone de-β-hydroxybutyrylation activities but displays no clear class selectivity, likely because the Zn-dependent HDACs do not require a β-staple conformation of peptidyl substrate for recognition^51^.

Histone acetyl as well as non-acetyl acylations are often linked to active gene transcription. Intriguingly, the establishment of histone acetyl/acylation states are likely hierarchical, which is reflected by the fact that many histone acetyl/acyl-transferases are often organized into large complexes that contain histone acylation reader modules^52^. For example, our recent work on YEATS2-containting ATAC acetyltransferase complex revealed a “read-write” Kac signal amplification mechanism in which recognition of H3K27ac by YEATS2 is required for ATAC-dependent maintenance of H3K9ac, thus ensuring full activation of ribosomal genes through establishment of histone hyperacetylation^53^. Here we revealed that downregulation of histone acylation levels by sirtuin family members are also hierarchical, indicative of a mechanism of hierarchical gene repression. Conceivably, those acylation sites that survive sirtuin treatment may serve as cis-acting marks to avoid unwanted chromatin compaction; on the other hand, they may act as seeds for re-establishment of a hyper-acylation pattern for gene reactivation by recruiting downstream effector complexes. Such a mechanism is supported by the existence of glycine-flanking motif-specific readers, such as the Brdt bromodomain that recognizes H4 K5acK8ac acetylation patterns^54,55^.

In summary, we demonstrate that the sirtuin family member SIRT3 is an eraser of Kbhb with class-selectivity against non-β (e.g., glycine-flanking) sequence motifs. Our work on hierarchical histone deacylation by SIRT3 suggests a potential regulatory mechanism that connects metabolism to gene regulation through dynamic acylations in sirtuin and modification biology.

## Materials and methods

### Protein and peptide preparation

Human sirtuin genes are kind gifts from Dr. Jiahuai Han at Xiamen University. The NAD^+^-dependent deacetylase domain encompassing residues 118–399 of SIRT3 was subcloned into a pSUMOH10 vector (modified based on pET28b) containing an N-terminal 10xHIS-SUMO tag. All the mutants of SIRT3 were generated by the QuikChange site-directed mutagenesis strategy and verified by sequencing. Wild-type SIRT3_118–399_ was overexpressed in the *Escherichia coli* BL21 (DE3) strain (Novagen). After induction with 0.2 mM isopropyl β-d-thiogalactoside (IPTG) at 16 °C in LB medium supplemented with 0.1 mM ZnCl_2_ overnight, cells were harvested by centrifugation and suspended in buffer A (500 mM NaCl, 20 mM Tris-HCl, pH 7.5, 5% glycerol, 20 mM imidazole), then disrupted by an EmulsiFlex-C3 homogenizer (Avestin). The lysate was further cleared by centrifugation, and the supernatant was loaded onto a Histrap affinity column. After buffer A washing, bound proteins were subjected to on-column cleavage by ULP1 SUMO protease. The tag-free SIRT3_118–399_ protein was collected as flow-through, centrifuge-concentrated, and then purified by an anion-exchange QHP column followed by size exclusion chromatography on a Superdex 75 column (GE Healthcare). Purified peak fractions were pooled, concentrated, aliquoted, and stored at −80 °C for future use. All mutant SIRT3 and other sirtuin family proteins were purified using essentially the same procedure as described above.

All the histone peptides used in this study were synthesized at >95% purity by Beijing SciLight Biotechnology Ltd. Co.

### Isothermal titration calorimetry

Experiments were performed at 25 °C on a MicroCal PEAQ-ITC instrument (Malvern Instruments). The sample cell containing 200 μL of 50 μM protein was titrated with 17 successive injections of 750 μM peptide. Acquired titration curves were fitted with the Origin 7.0 program using the “one set of binding sites” binding model. Protein concentrations were measured based on the UV absorption at 280 nm. Peptide concentrations were measured by weighing in large quantity.

### In vitro deacylation assays

For deacylation activity profiling studies, the reaction system was prepared by mixing 5 μg of each sirtuin, 1 μg of each acylated lysine peptide and 5 mM NAD^+^ in a buffer solution containing 20 mM Tris-HCl, pH 7.5 and 1 mM DTT. For SIRT3 catalyzed deacylation assays, 1 μM SIRT3 and 100 μM of each histone Kbhb peptide were added into the same reaction solution. For enzymatic kinetics studies, 1 μM of SIRT3 was incubated with different concentrations of histone H3_1–15_K9bhb-S/R (20, 40, 60, 80, 100, 200, 300, 400, and 500 μM) in the same reaction buffer at 37 °C for a certain period of time within the initial linear range. After incubation, the reactions were stopped by adding trifluoroacetic acid (TFA) to a final concentration of 5% (v/v) followed by immediate frozen in liquid nitrogen. Enzyme-free reaction systems were used as controls. The resultant reaction mixtures were then analyzed either RP-HPLC or MALDI-TOF MS.

For RP-HPLC analysis, samples were analyzed by a Dionex/Thermo UltiMate 3000 HPLC system with an Acclaim^TM^ RSLC 120 C18 column (2.1 mm × 100 mm, 2.2 μm). After loading, the reaction mixtures were washed by buffer A (0.1% TFA in water) for 10 min, and then eluted by a gradient of 1–20% buffer B (0.1% TFA in acetonitrile) over 20 min. The flow rate was 0.4 mL/min, and the wavelength for UV detection was 215 nm. For mass spectrometry, samples were analyzed by a 4800 plus MALDI TOF/TOF Analyzer (Applied Biosystems/MDS SCIEX) and operated in the Reflector Positive mode using 4000 Series Explorer Software. Laser intensity was set up to 3500 V.

For dot-blot deacylation experiment, 500 ng histone peptide H3_1–15_K9bhb was firstly added to nitrocellulose (NC) membrane, which is then air-dried and incubated with SIRT3-containing solution for 2 h at 37 °C. SIRT3 were prepared as a concentration gradient (0, 0.01, 0.05, 0.1, 0.5, and 10 μM) in reaction buffer 100 mM NaCl, 20 mM Tris-HCl, pH 7.5, 1 mM DTT, and 5 mM NAD^+^. After reaction, anti-H3K9bhb antibody (PTM Biolabs) was used to monitor the residue H3K9bhb on the membrane.

For nucleosome-based de-β-hydroxybutyrylation assays, Kbhb-modified nucleosomes were prepared using bhb-treated HEK293T cells, and then are subjected for SIRT3 treatment. The reaction system was prepared by mixing 1 μg/μL wild type SIRT3 or its catalytic mutant H248Y, 1 μg/μL extracted nucleosomes and 5 mM NAD^+^ in a buffer solution containing 20 mM Tris-HCl, pH 7.5, 350 mM NaCl and 1 mM DTT. After incubation at 37 °C for 1 h, the reaction was stopped by adding 5× loading buffer, then boiled at 100 °C for 10 min. Enzyme-free reaction systems were used as controls. The resultant reaction mixtures were then analyzed by immunoblotting assays.

### Crystallization, data collection, and structure determination

Crystallization was performed via the sitting drop vapor diffusion method under 18 °C by mixing 1 μL protein with 1 μL reservoir solution. Wild-type SIRT3_118–399_ (8.7 mg/mL) was premixed with H3_6–15_K9bhb, H3_1–15_K4bhb, or H4_11–25_K16bhb peptide in a 1:20 molar ratio in buffer 100 mM NaCl, 20 mM Tris-HCl, pH 7.5, and 2 mM DTT. Complex crystals were obtained in the reservoir solutions 0.1 M MES pH 6.5, 25% PEG 1000 for SIRT3-H3_6–15_K9bhb, 0.2 M Li_2_SO_4_, 0.1 M sodium citrate, pH 6.0, 19% PEG 3350 for SIRT3-H3_1–15_K4bhb, and 0.1 M Bicine, pH 9.0, 10% PEG 6000 for SIRT3-H4_11–25_K16bhb, respectively.

For data collection, crystals were flash-frozen in liquid nitrogen under cryoprotectant solutions, which are corresponding reservoir solutions supplemented with 10% glycerol for SIRT3-H3_1–15_K4bhb and SIRT3-H3_6–15_K9bhb, and 25% ethylene glycol for SIRT3-H4_11–25_K16bhb. Diffraction data were collected at beamline BL17U1 at Shanghai Synchrotron Radiation Facility at 0.9792 Å. The diffraction data were indexed, integrated, and merged using the HKL2000 software package^56^. The complex structures were solved by molecular replacement using Molrep^57^ from the CCP4 suite with the H3K4cr-bound SIRT3 structure (PDB: 4V1C) as the search model. Refinement and model building were performed with PHENIX^58^ and COOT^59^, respectively. The data collection and structure refinement statistics are summarized in Table S2.

### Immunoblotting

HEK293T cells were collected and boiled in RIPA lysis buffer (ThermoFisher) in the presence of NAM, TSA, and protease inhibitor cocktail (Selleck). Proteins in the lysate were then separated by SDS-PAGE and transferred onto a NC membrane for blotting using annotated anti-Kbhb or anti-histone antibodies (PTM Biolabs). A dilution factor of 1:2000 was used for most primary antibodies. The catalog numbers of antibodies used in this study are listed: anti-H3K4bhb: PTM-1258, anti-H3K9bhb: PTM-1250, anti-H3K14bhb: PTM-1251, anti-H3K18bhb: PTM-1292, anti-H3K23bhb: PTM-1300, anti-H4K5bhb: PTM-1205, anti-H4K8bhb: PTM-1253, anti-H4K12bhb: PTM-1206, anti-H4K16bhb: PTM-1262, anti-H3: PTM-1001, and anti-H4: PTM-1003.

### Co-cocalization analysis

HEK293T cells were transfected with plasmids encoding human sirtuins (SIRT1-7) with C-terminal EGFP using the Lipofectmine 2000 reagent (Invitrogen). After 48 h of transfection, cells were fixed with 4% (w/v) paraformaldehyde in PBS for 10 min, permeabilized with 0.2% Triton X-100 at room temperature for 5 min, and washed with PBS. Nuclei were counterstained with DAPI Fluoromount-G (Southern Biotech, 0100-20). Fluorescent images were taken with a Zeiss710 confocal laser scanning microscope system. Projected images were generated by ZEN 2 lite software (Carl Zeiss).

### Subcellular fractionation analysis

HEK293T cells were harvested by centrifugation and washed with PBS. All fractions were extracted for immunoblotting assays using Subcellular Protein Fractionation Kit for Cultured Cells (Thermo Scientific).

### Accession codes

The atomic coordinates and structure factors of SIRT3_118–399_-H3_6–15_K9bhb, H3_1–15_K4bhb, and H4_11–25_K16bhb have been deposited in Protein Data Bank under accession codes 5Z93, 5Z94, and 5ZGC.

## Supplementary information

Supplementary Information

Supplemental Table S1

Supplemental Movie S1

Supplemental Movie S2

## Supplementary information

**Supplementary Information** accompanies the paper at (10.1038/s41421-019-0103-0).
