# Supplementary material for: Molecular basis for hierarchical histone de-β-hydroxybutyrylation by SIRT3
Source: Cell Discov. 2019 Jul 9;5:35. doi: 10.1038/s41421-019-0103-0 (PMC6796883; doi:10.1038/s41421-019-0103-0)
Supplement: Supplementary file 1 — Supplementary Information [file 41421_2019_103_MOESM1_ESM.pdf]

Supplementary Information

Molecular basis for hierarchical histone de-β-hydroxybutyrylation by SIRT3

Xingrun Zhang[1], Ruili Cao[1], Jinrong Niu[1], Shumin Yang[1], Huida Ma[1], Shuai Zhao[1], and Haitao Li[1*]

[1]MOE Key Laboratory of Protein Sciences, Beijing Advanced Innovation Center for Structural Biology, Beijing Frontier Research Center for Biological Structure, Tsinghua-Peking Joint Center for Life Sciences, Department of Basic Medical Sciences, School of Medicine, Tsinghua University, Beijing 100084, China.

*Correspondence: lht@tsinghua.edu.cn

Supplementary Fig. S1: Architecture and SDS-PAGE analysis of purified human and E.coli sirtuin proteins.

Supplementary Fig. S2: ITC fitting curves between sirtuin proteins and acylated histone H3K9 peptides.

Supplementary Fig. S3: Summary of MALDI-TOF MS-based deacylation assays of human SIRT1-7 and bacterial CobB.

Supplementary Fig. S4: Structural analysis of S/R-Kbhb recognition by SIRT3 and ITC titrations monitoring wild type and mutant SIRT3-H3K9bhb interactions.

Supplementary Fig. S5: Binding affinity and deacylation activity of SIRT3 on R-form Kbhb histone peptides.

Supplementary Fig. S6: Michaelis–Menten plots showing the enzymatic kinetics of SIRT3-catalyzed deacyaltion reaction of histone S-form Kbhb peptides.

Supplementary Fig. S7: HDAC3 catalyzes hydrolysis of histone Kbhb with broad activities *in vitro*.

Supplementary Fig. S8: Specificity of H3K9bhb antibody as revealed by dot-blot assay.

Supplementary Fig. S9: Fluorescent and immunoblotting studies of human sirtuin family members in HEK293T cells.

Supplementary Fig. S10: Structure-based sequence alignment of human sirtuins and bacterial CobB.

Supplementary Table S2: Data collection and refinement statistics.

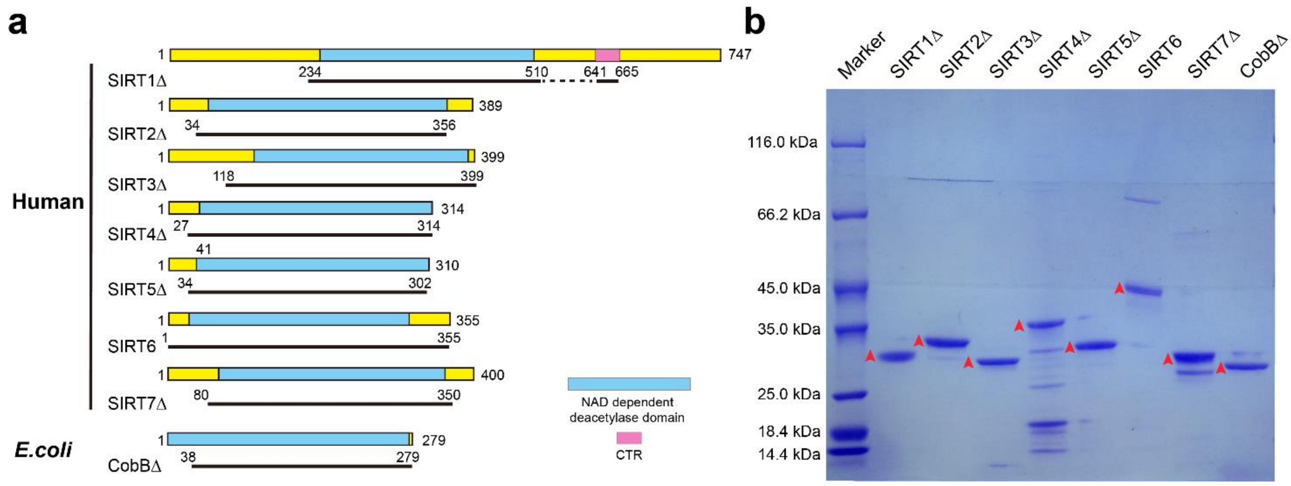

Supplementary Fig. S1 Architecture and SDS-PAGE Analysis of Purified Human and *E.coli* Sirtuin Proteins. (a) Schematic diagram of sirtuins protein constructs purified *in vitro*. NAD^+-dependent deacetylase domains are colored in cyan. C-terminal regulatory region in SIRT1 is colored in magenta. (b) Coomassie blue staining SDS-PAGE results of purified Sirtuin proteins. Targeted protein bands are annotated by red arrows.

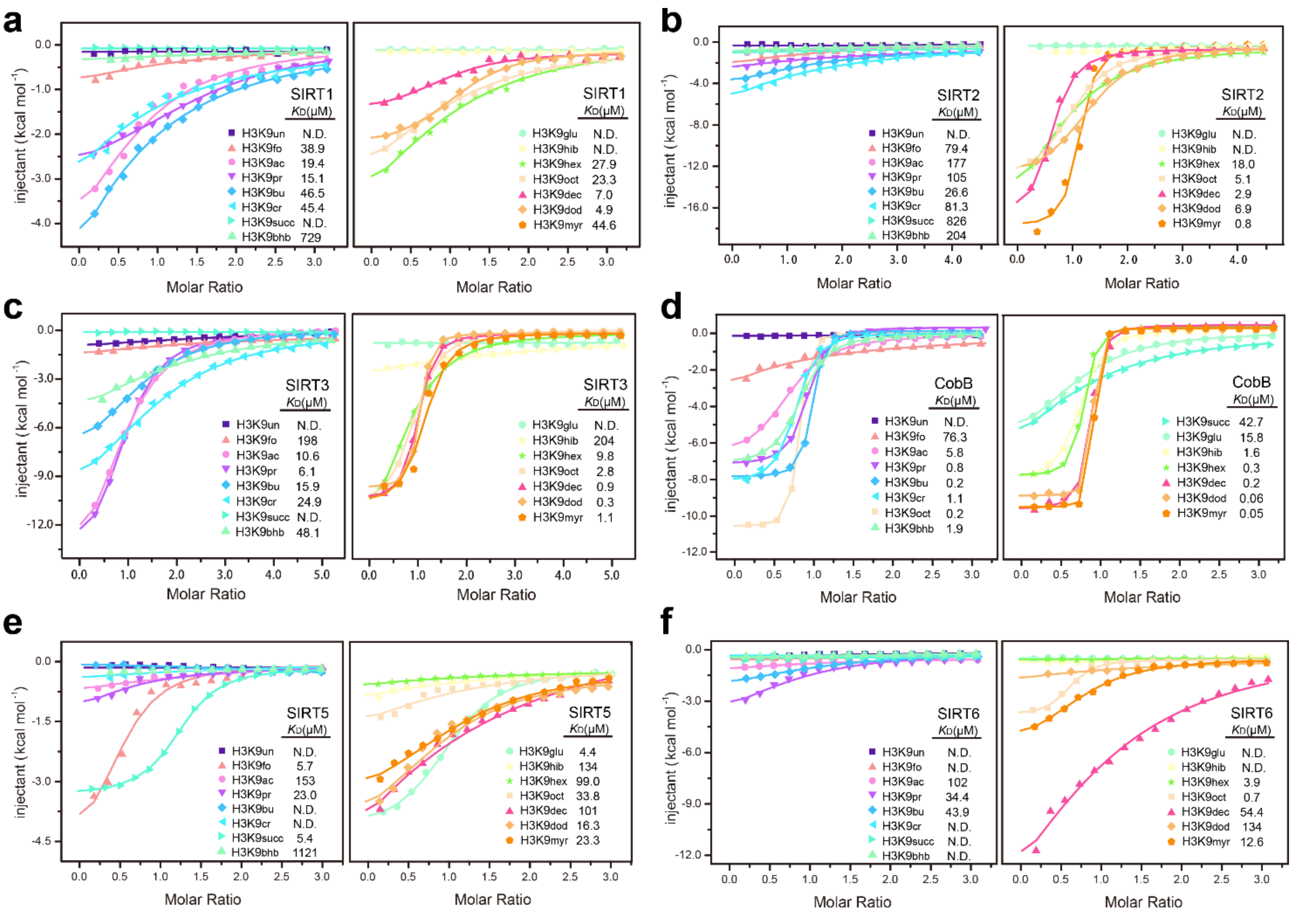

Supplementary Fig. S2 ITC Fitting Curves between Sirtuin Proteins and Acylated Histone H3K9 Peptides. ITC fitting curves of SIRT1 (a), SIRT2 (b), SIRT3 (c), SIRT5 (e), SIRT6 (f) and CobB (d) titrated with different acylated lysine on $H3_{1\text{-}15}K9$ peptides.

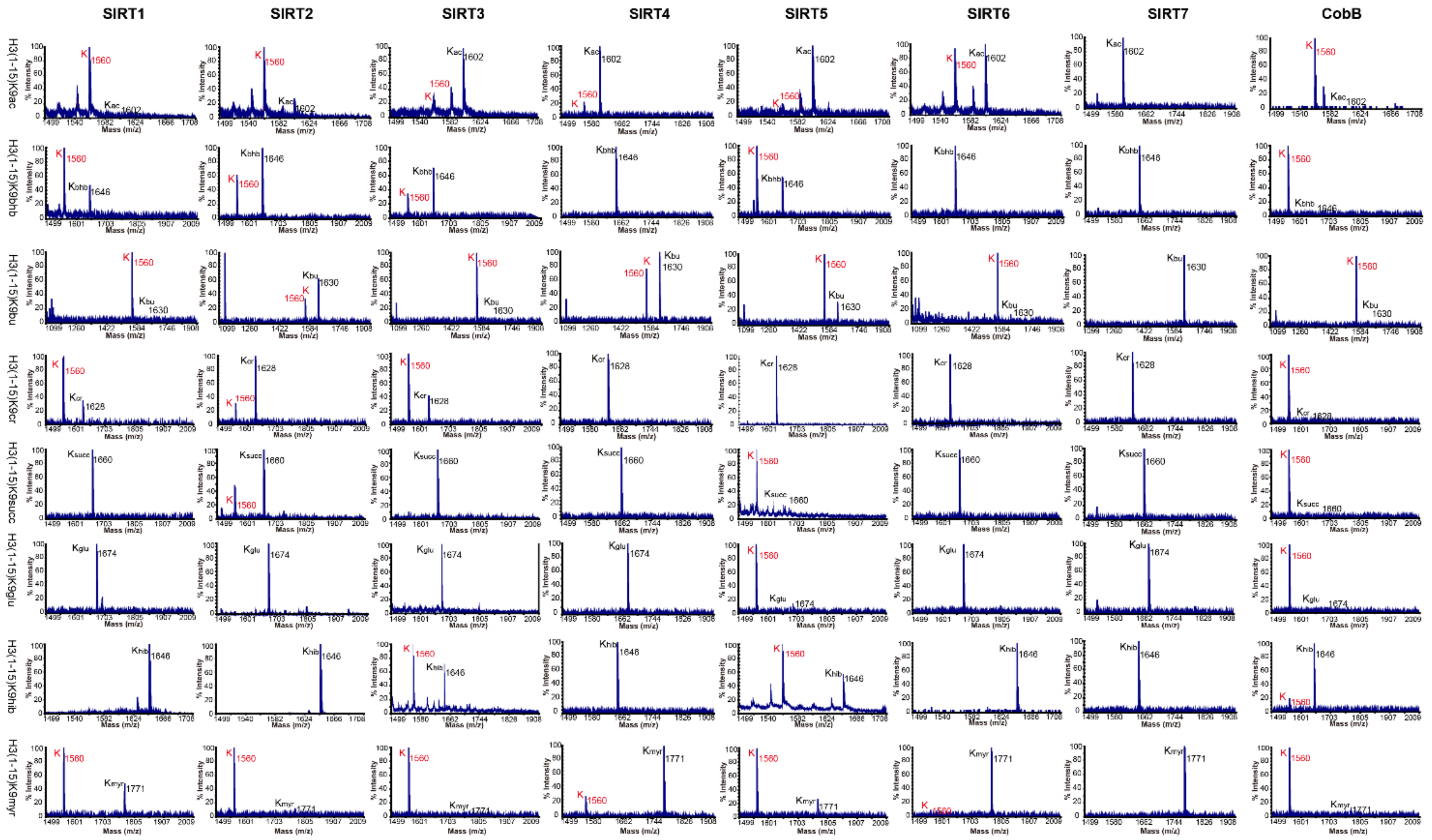

Supplementary Fig. S3 Summary of MALDI-TOF MS-based Deacylation Assays of Human SIRT1-7 and Bacterial CobB. Peaks with red labels: deacylated H3$_{1-15}$K9 products; Peaks with black labels: acylated H3$_{1-15}$K9 substrates. The MALDI-TOF MS assays were not performed for

quantitative comparison of the enzymatic efficiency

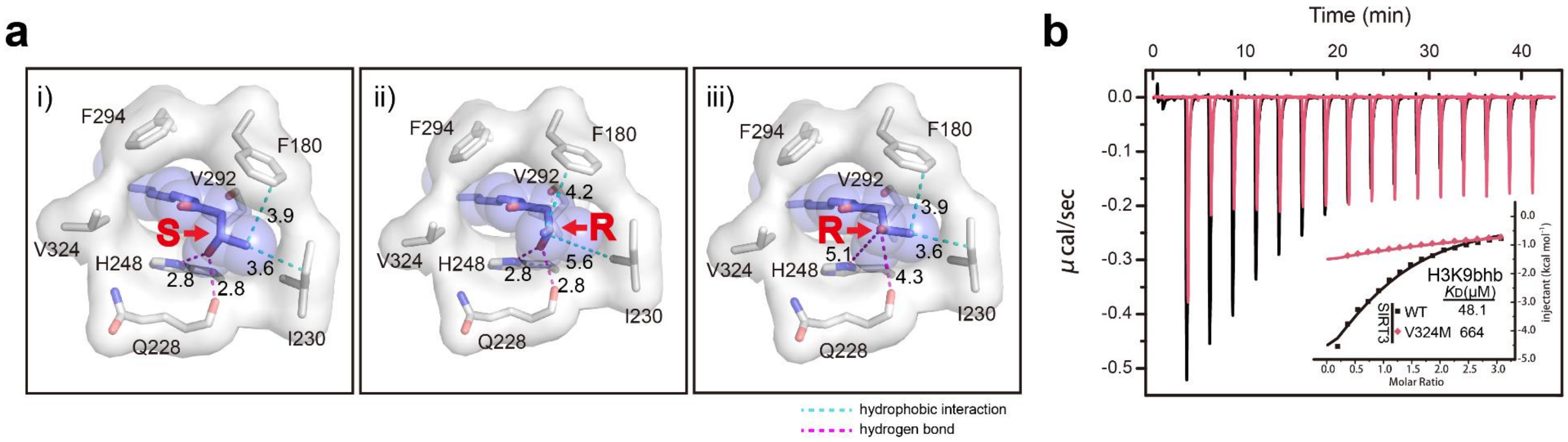

Supplementary Fig. S4 Structural Analysis of S/R-Kbhb Recognition by SIRT3 and ITC Titrations Monitoring Wild Type and Mutant SIRT3-H3K9bhb Interactions. (a) Structural analysis of Kbhb-SIRT3 catalytic pocket interaction using S-Kbhb (i) or R-Kbhb (3'-OH, ii or 4'-C, iii aligned with S-Kbhb). Distances of intermolecular interactions are measured and indicated in the figure. (b) ITC titration and fitting curves of wild-type (black) or V324 mutant (red) SIRT3 with $H3_{1-15}$K9bhb peptide synthesized as a racemic mixture.

a

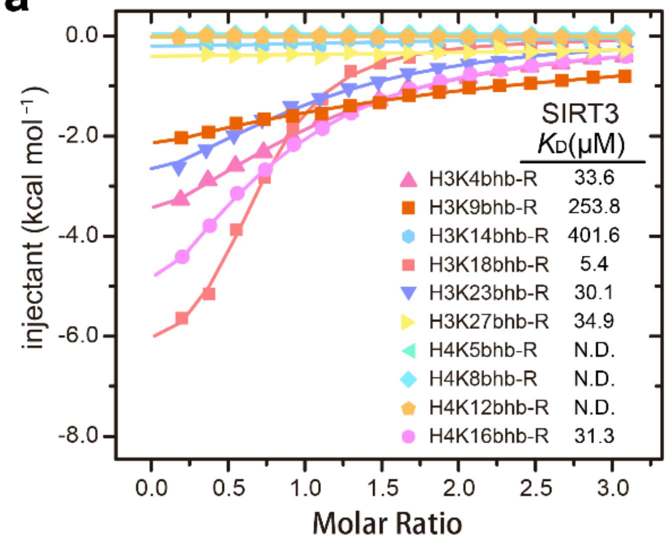

b

Kbhb-R	H3K4	H3K9	H3K14	H3K18	H3K23	H3K27	H4K5	H4K8	H4K12	H4K16
SIRT3	●●	●	●	●	●●	●	N.D.	N.D.	N.D.	●●
HDAC3	√	√	√	√	√	√	√	√	√	√

Supplementary Fig. S5 Binding Affinity and Deacylation Activity of SIRT3 on R-form Kbhb Histone Peptides. (a) ITC fitting curves of SIRT3 titrated with all histone R-form Kbhb peptides. (b) Summary of deacylation activities over all histone R-formed Kbhb peptides catalyzed by SIRT3 (detected by RP-HPLC) and HDAC3 (detected by MALDI-TOF MS). N.D., not detected. Numbers of black dots denote the relative activities as reflected by the abundance of the deacylated products. ●, 0-2% product yield; ●●, 2%-4% product yield; ●●●, 4%-6% product yield.

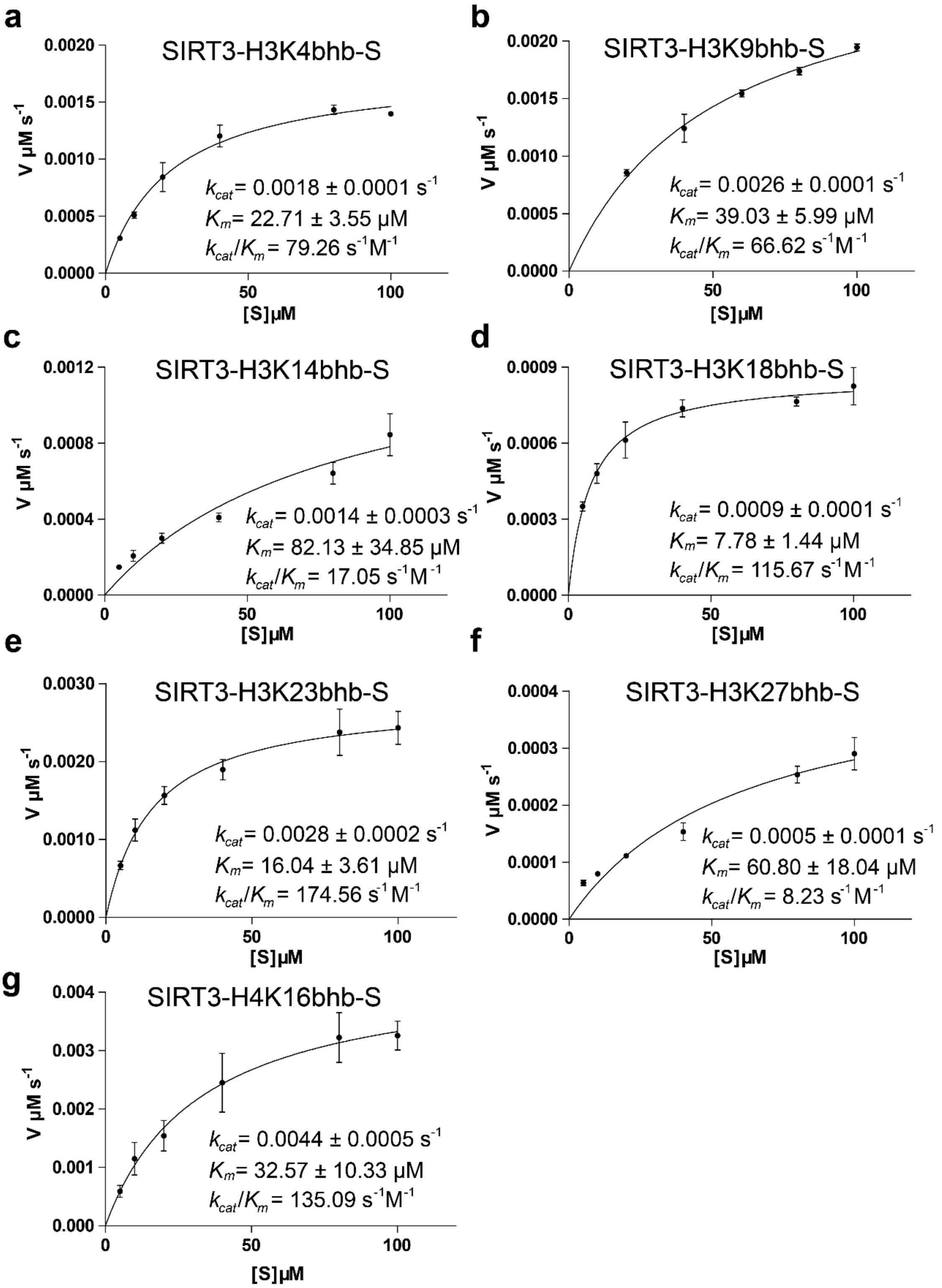

Supplementary Fig. S6 Michaelis–Menten Plots Showing the Enzymatic Kinetics of SIRT3-catalyzed Deacyaltion Reaction of Histone S-form Kbhb Peptides. Enzymatic kinetics studies against H3$_{1-15}$ K4bhb-S (a), K9bhb-S (b), H3K$_{7-21}$14bhb-S (c), H3K$_{11-25}$18bhb-S (d), H3$_{16-30}$K23bhb-S(e), H3K$_{21-35}$27bhb (f), and H4$_{11-25}$K16bhb (g) peptide substrates. Experiments were performed in triplicates and the kinetic parameters are reported as mean ± SEM (n=3).

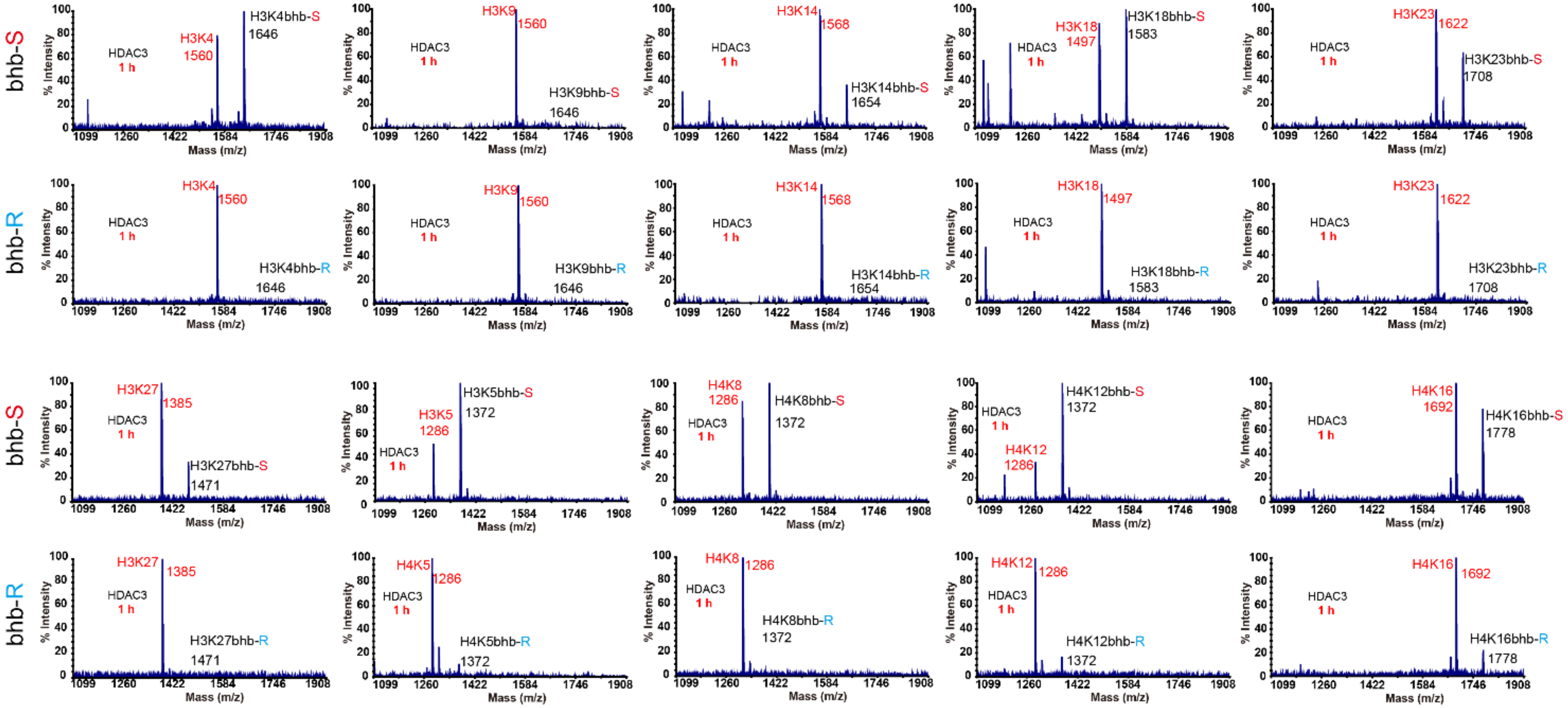

Supplementary Fig. S7 HDAC3 Catalyzes Hydrolysis of Histone Kbhb with Broad Activities *in vitro*. Deacylation activity of HDAC3 on S- and R-formed H3$_{1\text{-}15}$K4bhb, H3$_{1\text{-}15}$K9bhb, H3$_{7\text{-}21}$K14bhb, H3$_{11\text{-}25}$K18bhb, H3$_{16\text{-}30}$K23bhb, H3$_{21\text{-}35}$K27bhb, H4$_{1\text{-}15}$K5bhb, H4$_{1\text{-}15}$K8bhb, H4$_{1\text{-}15}$K12bhb, and H4$_{11\text{-}25}$K16bhb substrates as revealed by MALDI-TOF MS. Peaks with red labels: deacylated products; Peaks with black labels: acylated substrates.

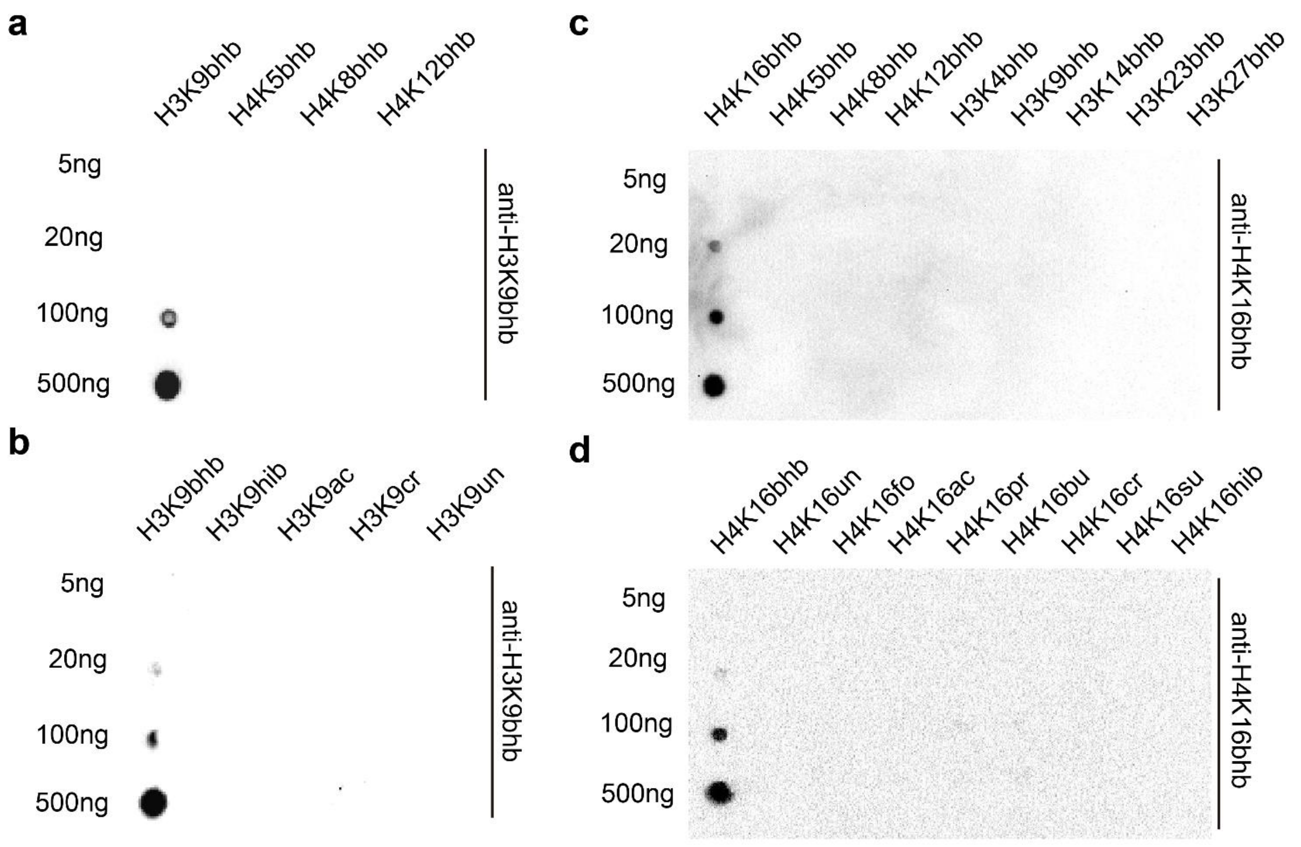

Supplementary Fig. S8 Specificity of H3K9bhb Antibody as Revealed by Dot-blot Assay.
(a, b) Site-(a) and type-(b) specificity of H3K9bhb antibody revealed by dot blot assays. (c, d) Site-(c) and type-(d) specificity of H4K16bhb antibody revealed by dot blot assays.

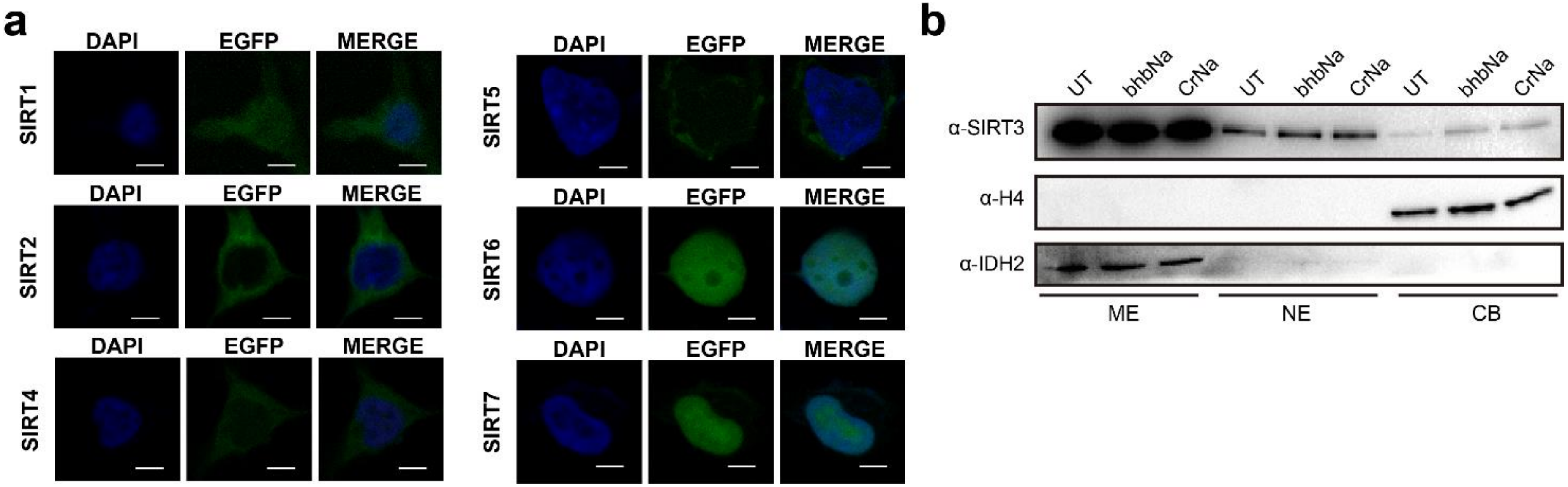

Supplementary Fig. S9 Fluorescent and Immunoblotting Studies of Human Sirtuin Family Members in HEK293T Cells. (a) Fluorescent analysis of other human sirtuins. Scale bars, 5 μm. (b) Immunoblotting results of SIRT3 in each subcellular fractionation of HEK293T cells. UT, untreated; bhbNa, 10 mM bhbNa treated; CrNa, 10 mM treated. ME, membrane extracts; NE, soluble nuclear extracts; CB, chromatin-bound extracts.

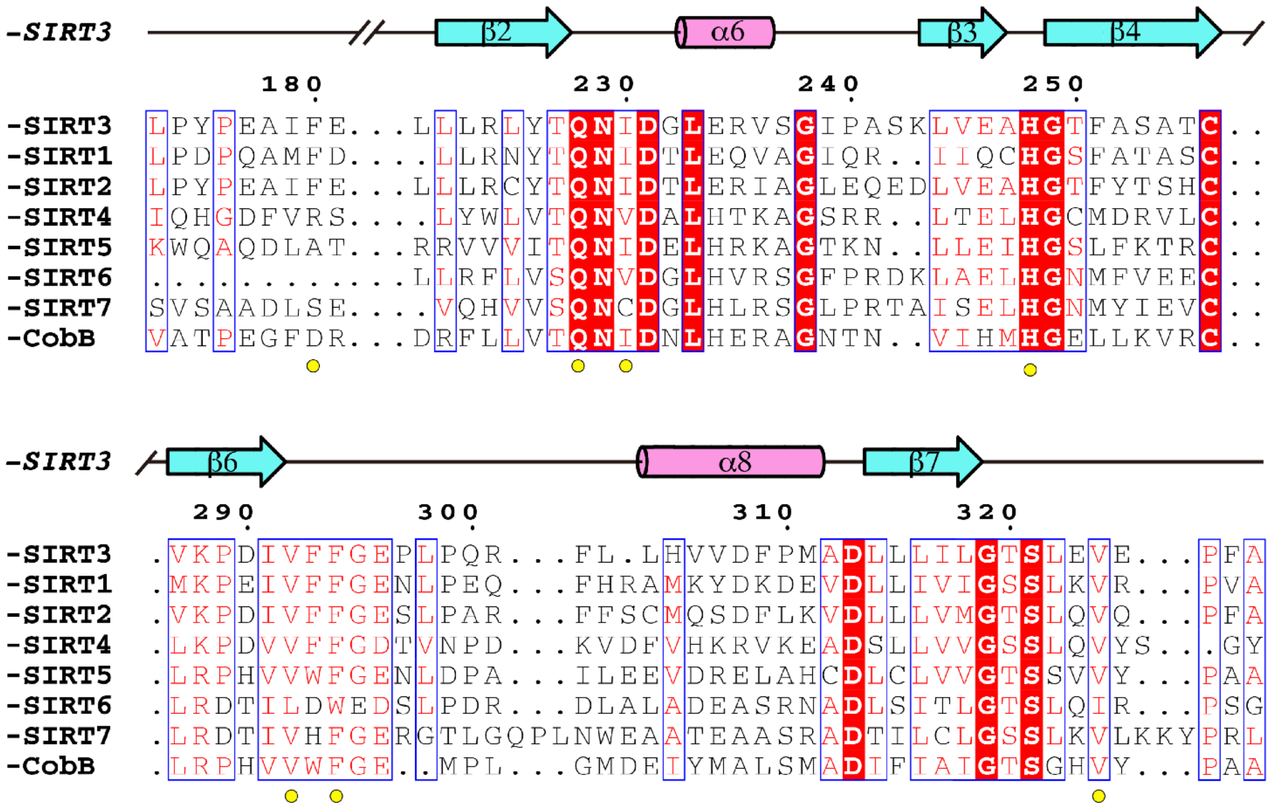

Supplementary Fig. S10 Structure-based Sequence Alignment of Human Sirtuins and Bacterial CobB. Key residues that participate in H3K9bhb recognition are highlighted with yellow circles.

Supplementary Table S2: Data collection and refinement statistics.

Crystal	SIRT3-H3$_{1-15}$K4bhb	SIRT3-H3$_{6-15}$K9bhb	SIRT3-H4$_{11-25}$K16bhb
Data Collection			
Space group	$P2_1$	$P2_12_12_1$	$P4_32_12$
Unit Cell			
a, b, c (Å)<br>α, β, γ (°)	75.5, 76.3, 75.4<br>90, 115.8, 90	33.4, 86.0, 88.4<br>90, 90, 90	138.5, 138.5, 242.9<br>90, 90, 90
Resolution (Å)	50-1.90 (1.93-1.90)*	50-1.95 (1.98-1.95)	50-2.90 (2.97-2.90)
R_{sym} (%)	7.9 (54.2)	8.4 (61.3)	10.0 (118.8)
R_{pim} /CC1/2 (%)	4.8 (32.6) / (80.9)	3.7 (28.5) / (82.3)	3.9 (47.0) / (70.8)
I/σ (I)	26.4 (2.8)	24.7 (2.3)	13.6 (1.2)
Completeness (%)	99.8 (100.0)	99.2 (97.5)	99.6 (99.4)
Redundancy	3.7 (3.7)	6.2 (5.4)	7.5 (6.8)
Refinement (F>0)			
Resolution (Å)	37.6-1.90	31.2-1.95	43.2-2.90
No. of unique reflections	60,593	19,211	52,894
R_{work}/R_{free} (%) [c]	16.1/19.1	16.1/20.9	20.1/26.6
No. of non-H atoms			
Protein	4,308	2,152	12,019
Peptide	118	52	351
Water	507	222	61
Zn^{2+}	2	1	5
Average B-factors (Å^2)			
Protein	33.4	35.0	78.6
Peptide	43.9	44.0	83.2
Water	40.2	41.3	55.2
Zn^{2+}	20.0	26.8	102.7
RMSD bonds (Å)	0.003	0.010	0.010
RMSD angle (°)	0.628	1.000	1.231

* Highest resolution shell is shown in parenthesis.
